# Supplementary material for: Qualitative assessment of the impact of socioeconomic and cultural barriers on uptake and utilisation of tuberculosis diagnostic and treatment tools in East Africa: a cross-sectional study
Source: BMJ Open. 2021 Jul 11;11(7):e050911. doi: 10.1136/bmjopen-2021-050911 (PMC8276309; doi:10.1136/bmjopen-2021-050911)
Supplement: Supplementary data [file bmjopen-2021-050911supp001.pdf]

Tuberculosis: Working together to Empower Nations' Diagnostic Effort (TWENDE)

Assessing the coverage and usage of Xpert MTB/RIF & Line Probe Assay services in Tanzania, Kenya and Uganda

Interview Guide for Focus Groups & Focus Group Discussions

1.1 Opening Lines (after informed consent has been reviewed and participant has agreed)

In this Focus Group Discussion, I would like to hear your views about the '*stigma related to tuberculosis'; 'diagnosis and treatment of tuberculosis'*. You are an expert in this subject and so feel free to share your views among each other however you wish. I may minimally interrupt your discussion and ask some questions which are coming from your comments, which are interested in order to move the discussion along, but will try and keep my interruptions very minimum. Please feel free to elaborate on anything you feel is important for me to know about. I am interested in all what you consider the negative and positives in diagnosis and treatment also what you perceive as a stigma on TB in Tanzania. Remember, there is no any right or wrong answers and your contributions are so valuable. Please feel free to discuss openly and freely about what you know on the state of stigma, diagnosis and treatment of TB among self, family, community, and national here in Tanzania.

1.2 Initial Identifying Statement of the Facilitator

So let us begin [*press play on audio recorder*]: "It is [*date*] and I am sitting in [*place*] with [*participant's group name*]. We are going to converse about their views about the topic of ''stigma *related to tuberculosis''; ''diagnosis and treatment of TB''* in [name district].

1.3

- In your experiences, how accessible are TB diagnostic laboratories and treatment centres in [name district]?
    - [*probe*] In your opinions, in general, what is the state of TB diagnosis and treatment services in [name district]?
    - [*Probe*]When do you go for your check-ups when you feel you have TB symptoms?
    - [*Probe*]Do you pay for TB diagnosis or treatment?
      [*Probe*]What are your recommendations, to make things better in terms of TB diagnosis and treatment services in [name district]?
- In your experiences, are TB patients in general stigmatised here in your community in [name ethnic group, village, district, region, Tanzania]?
    - If yes, how so? Why do you think so?
    - [P*robe*] What is your recommendation on how stigmatisation of TB patients may be reduced?

Msoka EF, *et al. BMJ Open* 2021; 11:e050911. doi: 10.1136/bmjopen-2021-050911

1.4 Conclusion of Interview

Thank you so much group members for taking the time to discuss with me today. I learned a great deal from this discussion, and I really appreciate your willingness to share your views, insights and opinions with me and by extension the TWENDE project.

[*Allow respondents to comment if he wishes.*]

Msoka EF, *et al. BMJ Open* 2021; 11:e050911. doi: 10.1136/bmjopen-2021-050911
