# Supplementary material for: Qualitative assessment of the impact of socioeconomic and cultural barriers on uptake and utilisation of tuberculosis diagnostic and treatment tools in East Africa: a cross-sectional study
Source: BMJ Open. 2021 Jul 11;11(7):e050911. doi: 10.1136/bmjopen-2021-050911 (PMC8276309; doi:10.1136/bmjopen-2021-050911)
Supplement: Author's manuscript [file bmjopen-2021-050911.draft_revisions.pdf]

BMJ Open is committed to open peer review. As part of this commitment we make the peer review history of every article we publish publicly available.

When an article is published we post the peer reviewers' comments and the authors' responses online. We also post the versions of the paper that were used during peer review. These are the versions that the peer review comments apply to.

The versions of the paper that follow are the versions that were submitted during the peer review process. They are not the versions of record or the final published versions. They should not be cited or distributed as the published version of this manuscript.

BMJ Open is an open access journal and the full, final, typeset and author-corrected version of record of the manuscript is available on our site with no access controls, subscription charges or pay-per-view fees (http://bmjopen.bmj.com).

If you have any questions on BMJ Open's open peer review process please email info.bmjopen@bmj.com

BMJ Open

Impact of socio-economic and cultural barriers on uptake and utilisation of diagnostic and treatment tools into policy and practice in East Africa- A qualitative study

Journal:	*BMJ Open*
Manuscript ID	bmjopen-2021-050911
Article Type:	Original research
Date Submitted by the Author:	07-Mar-2021
Complete List of Authors:	Msoka, Elizabeth ; Kilimanjaro Christian Medical University College, Clinical Trial<br>Orina, Fred; Kenya Medical Research Institute<br>Sanga, Erica; NIMR-Mbeya Medical Research Programme, Social Science<br>Miheso, Barbara; Kenya Medical Research Institute<br>Mwanyonga, Simeon; NIMR-Mbeya Medical Research Programme<br>Meme, Helen; Kenya Medical Research Institute<br>Kiula, Kiula; Kilimanjaro Christian Medical Centre; The University of Dodoma<br>Liyoyo, Alphonce; Kilimanjaro Christian Medical Centre<br>Mwebaza, Ivan; Makerere University<br>Aturinde, Augustus; Lund University, Department of Physical Geography and Ecosystem Science; Kyambogo University, Department of Physical Geography and Ecosystem Science<br>Joloba, Moses; Makerere University College of Health Sciences<br>mmbaga, blandina; Kilimanjaro Christian Medical University College, ; Kilimanjaro Clinical Researxh Institute<br>Amukoye, Evans; Kenya Medical Research Institute<br>Ntinginya, Nyanda; NIMR-Mbeya Medical Research Programme<br>Gillespie, Stephen; University of St Andrews<br>Sabiiti, Wilber; University of St Andrews, School of Medicine
Keywords:	Diagnostic microbiology < INFECTIOUS DISEASES, Molecular diagnostics < INFECTIOUS DISEASES, Health policy < HEALTH SERVICES ADMINISTRATION & MANAGEMENT, Tuberculosis < INFECTIOUS DISEASES, Public health < INFECTIOUS DISEASES

SCHOLARONE™
Manuscripts

I, the Submitting Author has the right to grant and does grant on behalf of all authors of the Work (as defined in the below author licence), an exclusive licence and/or a non-exclusive licence for contributions from authors who are: i) UK Crown employees; ii) where BMJ has agreed a CC-BY licence shall apply, and/or iii) in accordance with the terms applicable for US Federal Government officers or employees acting as part of their official duties; on a worldwide, perpetual, irrevocable, royalty-free basis to BMJ Publishing Group Ltd ("BMJ") its licensees and where the relevant Journal is co-owned by BMJ to the co-owners of the Journal, to publish the Work in this journal and any other BMJ products and to exploit all rights, as set out in our licence.

The Submitting Author accepts and understands that any supply made under these terms is made by BMJ to the Submitting Author unless you are acting as an employee on behalf of your employer or a postgraduate student of an affiliated institution which is paying any applicable article publishing charge ("APC") for Open Access articles. Where the Submitting Author wishes to make the Work available on an Open Access basis (and intends to pay the relevant APC), the terms of reuse of such Open Access shall be governed by a Creative Commons licence – details of these licences and which Creative Commons licence will apply to this Work are set out in our licence referred to above.

Other than as permitted in any relevant BMJ Author's Self Archiving Policies, I confirm this Work has not been accepted for publication elsewhere, is not being considered for publication elsewhere and does not duplicate material already published. I confirm all authors consent to publication of this Work and authorise the granting of this licence.

For peer review only

Impact of socio-economic and cultural barriers on uptake and utilisation of diagnostic and treatment tools into policy and practice in East Africa- A qualitative study

Elizabeth F. Msoka[1*,] MSc, Fred Orina[2*] MSc, Erica Sanga[3]PhD, Barbara Miheso[2]BSc, Simeon Mwanyonga[4]BA, Hellen Meme[2]MD, Kiula Kiula[5]MA, Aliphonce Liyoyo[,6]MSc, Ivan Mwebaza[7]MSc, Augustus Aturinde[8,9]MSc, Moses Joloba[7]PhD, Blandina Theophil Mmbaga[1]PhD, Evans Amukoye[2]MD, Nyanda Elias Ntinginya[4] PhD, Stephen H Gillespie[11]DSc, Wilber Sabiiti[11]PhD

*Contributed equally for the 1st author position.

Author Affiliations

1. Kilimanjaro Christian Medical Centre, Moshi, Tanzania
2. Kenya Medical Research Institute (KEMRI), Nairobi, Kenya.
3. National Institute of Medical Research-Mwanza Medical Research Centre, Mwanza, Tanzania
4. National Institute of Medical Research - Mbeya Medical Research Centre (NIMR-MMRC), Mbeya, Tanzania.
5. Institute of Development studies, University of Dodoma, Dodoma, Tanzania.
6. Kibong'oto Infectious Diseases Hospital, Mae Street, Lomakaa road, Sanya Juu, Siha Kilimanjaro, Tanzania
7. School of Biomedical Sciences, College of Health Sciences, Makerere University, Kampala, Uganda.
8. Department of Physical Geography and Ecosystem Science, Lund University, Sweden.
9. Department of Lands and Architectural Studies, Kyambogo University, Kampala, Uganda.
10. School of Biomedical Sciences, College of Health Sciences, Makerere University, Kampala, Uganda.
11. School of Medicine, University of St Andrews, St Andrews, United Kingdom.

Corresponding Author:
Elizabeth Francis Msoka
Kilimanjaro Clinical Research Institute
P.O.BOX 2236
Kilimanjaro-Moshi, Tanzania
Email: e.fbright@kcri.ac.tz
Tel +255(27)2754201

For peer review only

For peer review only - http://bmjopen.bmj.com/site/about/guidelines.xhtml

Abstract

Objectives: Early diagnosis and timely treatment are key elements of a successful healthcare system. We assessed the role of socioeconomic and cultural norms in accelerating or decelerating uptake and utilisation of health technologies into policy and practice.

Setting: Secondary and tertiary level healthcare facilities in three East African countries. Level of healthcare facility (HCF) was selected based on the WHO recommendation for implantation of tuberculosis (TB) molecular diagnostics.

Participants: Using implementation of TB diagnostics as a model, we purposively selected participants (TB patients, carers, survivors, healthcare practitioners, community members, opinion leaders and policymakers) based on their role as stakeholders. In-Depth Interviews (IDIs), Key Informant Interviews and Focus Group Discussions (FGDs) were held to collect the data between 2016-18. The data were transcribed, translated, coded and analysed by thematic-content analysis.

Results: A total of 712 individuals participated in the study. Socioeconomic and cultural factors such as poverty, stigma, and inadequate knowledge about causes of disease and available remedies, cultural beliefs were associated with low access and utilisation of diagnostic and treatment tools for tuberculosis. Poverty made people hesitate to seek formal healthcare resulting in delayed diagnosis and resorting to self-medication and cheap herbal alternatives. Fear of stigma made people hide their sickness and avoid reporting for follow-up treatment visits. Inadequate knowledge and beliefs were fertile ground for aggravated stigma and believing that diseases like TB are caused by spirits and thus cured by spiritual rituals or religious prayers. Cultural norms were also the basis of gender-based imbalance in accessing care, "I could not go to hospital without my husband's permission", TB survivor.

Conclusion: Our findings show that socioeconomic and cultural factors are substantial "road blocks" to accelerating the uptake and utilisation of diagnostic and treatment tools. Resolving these barriers should be given equal attention as is to health system barriers.

Keywords: TB, Maximizing uptake, utilisation of diagnostic, treatment tools, policy and practice, impact, socioeconomic, cultural norms, East Africa.

Summary

A well-functioning health system is critical for effective uptake and utilisation of health technologies. However, health system malfunctioning is often attributed to barriers such as underfinancing, inadequate human resource and poor governance. Whilst these health system barriers are important, this study aimed to investigate the role played by socioeconomic-cultural barriers in uptake and utilisation of diagnostic and treatment tools. Findings show that socioeconomic-cultural challenges faced by communities, families and/or individuals play a significant role in the degree of uptake and utilisation of healthcare technologies, and should therefore be given equal attention when investing in healthcare solutions.

Strengths and limitations

- The paper brings out the voices of a diversity of stakeholders within the health system, substantiating the impact of socioeconomic - cultural barriers on access and utilisation of healthcare technologies.
- The paper presents data from three countries demonstrating the generalisability of the socioeconomic and cultural barriers in impeding uptake and utilisation of healthcare technologies. The information is useful for both policymakers and health technology developers globally.
- In line with other studies, the paper shows that socioeconomic and cultural barriers primarily limit access to healthcare and the ability to adhere to care of which the largest hinderance occurs at primary care point i.e. between feeling unwell and accessing diagnosis at the healthcare facility.

For peer review only - http://bmjopen.bmj.com/site/about/guidelines.xhtml

- The paper also highlights the importance of adequate access to information as a tool for fighting mis/disinformation in order to increase uptake and utilisation of healthcare technologies.
- The limitation of the paper is that it presents the views of 712 participants, a number that constitutes a tiny percentage of the population of the three study countries.

Introduction

Tuberculosis (TB) claims more than a million lives every year of which the poorest of the world are most affected[1–4]. Considerable technological advances have been made in the diagnosis and treatment of TB but they remain less accessible to the communities that need it the most[5–7]. Only two thirds of the global reported TB cases are confirmed by a laboratory test[2]
Tanzania, Uganda and Kenya are among the 30 countries with highest burden of tuberculosis as well as TB/HIV co-infection in the world[1]. The high tuberculosis burden is characterised by poor diagnostic capability where many of high burdened countries still depend on microscopy, a less sensitive tool for diagnosis[9–12]. However, there are novel technologies including the Xpert MTB/RIF and Line probe assay which are WHO approved for rapid detection of tuberculosis and drug resistance that have been found to be implementable[11,13].

Knowledge of disease, social and cultural issues have been highlighted as important factors in the patient diagnosis and treatment pathway of TB[14]. Studies conducted elsewhere regarding TB and social cultural barriers have revealed that there is a complex interplay between contextual factors and community understanding of the disease. Cultural beliefs about causality and treatment-seeking paths were often mentioned in conjunction with biomedical views[16]. There was a strong interface between TB and HIV in communities, and knowledge of TB is often confused with HIV, additionally HIV-related stigma has been extended to those living with TB[16]. The stigma of TB infection being related to HIV infection has shown to have an impact on patients seeking treatment and their adherence to treatment[17–19]. These cultural barriers have been reported in most African communities where TB infection is associated or linked to witchcraft, thus leading to delay in successful diagnosis and treatment of TB[15,20,21]. Interestingly, lack of access to media was highlighted as significantly associated with low tuberculosis knowledge[22].

Socioeconomic activities such as mining are reported high risk for communicable illness including tuberculosis and HIV/AIDS[20]. Population migration has been shown to facilitate transmission of TB as well as causing treatment disruptions [23] [24].

Taken together, it was not clear how the socioeconomic and cultural factors could affect the uptake and utilisation of diagnostic and treatment tools in a health system. We set out to investigate these factors and the ways they impede uptake of health technologies in order to inform models for resolution. We show that socioeconomic and cultural norms substantially limit access and utilisation to healthcare services and resolving them is fundamental to having a well-functioning health system.

Methods

This was a qualitative part of a larger mixed methods study assessing health system and wider community impediments and opportunities to unlock them in order to achieve effective uptake and utilisation of diagnostic and treatment tools. Implementation of WHO approved tuberculosis diagnostics was used as benchmark to evaluate the phenomenon. The study was conducted between Jan and Dec 2017 in Uganda, Kenya and Tanzania. At the time of the study, only Kenya was defined as a middle-income country, and it has now been joined by Tanzania in this classification. This means the Tanzania results in this study reflect a low-income country setting.

For peer review only - http://bmjopen.bmj.com/site/about/guidelines.xhtml

Study design and participants

This was a cross sectional study targeting TB- patients and survivors, care givers, general members of the community served by the healthcare facility (HCF), healthcare practitioners, opinion leaders, local government authorities and policy/decision makers at local and national levels. A purposeful sampling method was used to select participants based on these categories and also to ensure representation by age, gender and geographical location. A combination of in-depth interviews (IDI) and focus group discussions (FGDs) were used to generate data for this study. Participants who participated in the in-depth interviews were not the same as those who participated in the FGDs, the study recruited them differently. Participants for the FGDs were identified through the community lead who acted as an important gate keeper in accessing these groups of participants, as they knew the area very well and were able to help with identifying potential participants. In each selected administrative region, five FGDs were undertaken with 12 participants per group and ten in-depth interviews were performed. FGDs were meant to capture a collective opinion of issues concerning diagnosis and treatment of TB. In depth interview were to get a deeper opinion of individuals without interference or fear of others.

Data management and analysis

All the in-depth and key informant interviews and focus group discussions (FGDs) were conducted by a trained Qualitative Data Investigators who underwent a five-day training held in Kampala, Uganda, May 2016. All the data collection tools including questionnaires and interview guides were pretested and revised for clarity and accuracy prior to being used in the field. The interviews were conducted in Swahili in Kenya and Tanzania and/or English in Uganda, in the offices or health facilities depending on the group of respondents. All interviews were audio recorded with permission from the respondents. Collected data were transcribed verbatim from Swahili and translated to English before being analysed by thematic-content approach[25]. Data was coded and synthesized using both inductive and deductive techniques by the first author and reviewed by two other Qualitative Data Investigators for consistency to produce list of themes, theme models, and classification related to the themes[26]. In keeping with the measures advised by Barbour 2001 independently, two researchers read and re-read all focus group and interview data, recording the emergent themes. The process involved careful reading of the interview transcripts for pattern recognition which were later translated to categories, subcategories in order to draw the conclusions from the data meanwhile developing themes. Themes further analysed to identify subthemes. However, in the process where there was disagreement in the themes and subthemes developed, discussion took place until agreement to ensure trustworthy of the data was achieved[28].

Ethics approval and consent to participate

The study gained ethical approval from respective institutional and national ethics committees represented in the consortium. In the UK, approval was obtained from University Teaching and Research Ethics Committee of University of St Andrews (MD12073), Uganda: Makerere University Institutional Research Ethics Board (IRB) and National Council of Science and Technology (HS 2129), Kenya: KEMRI Scientific and Ethics Review Unit (KEMRI/RES/7/3/1) and Tanzania: KCRI and Mbeya and southern highlands zonal IRBs and National Health Research Ethics Committee (NatHREC) at NIMR headquarters (NIMR/HQ/R.8a/vol.IX/1317). In addition to ethics approvals, access permissions were sought and obtained from local government authorities and participants whom involved in the study.

Public involvement statement

This was a people centred study involving people at grassroots up to policymakers. The views presented in the paper are views of the community groups and individuals in the study countries. This qualitative paper is the voice of the people: patients, survivors, carers, opinion leaders and policy makers. Regional and national public and policymakers' gatherings were held to share the findings of the study and policy briefs submitted to respective ministries of health.

For peer review only - http://bmjopen.bmj.com/site/about/guidelines.xhtml

Results

Participants

A total of 712 participants above 18 years of age participated in this study of whom 70 % were females. 65 % were from rural areas. Nine categories of individuals participated including 38 Laboratory technicians, 37 medical administrators, 29 Political leaders, 42 TB patients and survivors, 36 key informants, 42 healthcare providers, 27 caregivers and 19 private practitioners in the private and public sectors. 108 focus group discussions (FGDs) were held among community members, TB patients and caregivers. 270 in-depth interview (IDI) including key informant interviews (KIs) were conducted [Table 1].

Respondents	Total number
Laboratory technicians	38
TB patients	32
TB survivors	10
TB patient care givers	27
Local government authorities	29
Medical administrators and DTLCs	37
Medical practitioners/clinic staff/DOT Nurses	42
Private practitioners	19
Community members, practitioners & policymakers in FGDs	445

Table 1: Categories of respondents that participated in the study. Community members participating in FGDs and interviews constituted the largest proportion of participants.

Participants came from thirteen regions and fourteen counties with high prevalence of TB and HIV. In Tanzania the study was conducted in the following thirteen regions: Kilimanjaro, Manyara, Tanga, Mwanza, Kagera, Kigoma, Singida, Mbeya, Dodoma, Dar-es-salaam, Mtwara, Rukwa and Katavi. In Kenya the study was conducted in the following fourteen counties: Taita, Taveta, Mombasa, Garrisa, Wajir, Meru, Marsabit, Makueni, Muranga, Nairobi, West Pokot, Bomet, Bongoma and Nyamira. In Uganda the study was conducted in the following regions and Sub regions including: Northern region in Karamoja, Lango, Acholi, and West Nile; Eastern region in Teso, Sebei, Bugisu, Bugwere, Samia, Bunyole, Jopathola and Busoga Sub-region of Uganda; Southern region in Buganda, Buuruuli and Kooki Sub-region of Uganda; Western region in Bunyoro, Rwenzori, Ankole and Kigezi sub-region of Uganda (Fig.1).

Interview and FGD outcomes

A total of 378 interviews and FGD transcripts were obtained. Following analysis, the responses were grouped into two thematic categories: socio-cultural and socioeconomic barriers, each containing sub-themes including: knowledge of TB, diagnosis and treatment, Size, taste, and number of pills and side effects of TB treatment and Work patterns and responsibilities**.**

For peer review only - http://bmjopen.bmj.com/site/about/guidelines.xhtml

Socio-cultural barriers

Knowledge of TB, diagnosis and treatment: Knowledge about TB disease, causes, recognition and interpretation of signs and symptoms, and treatment was found to be low in the community members with about 50 % of respondents having some basic knowledge of TB. The low knowledge of TB was more pronounced rural communities compared to their counterparts living in the urban areas. The statements below illustrate the low knowledge on TB disease.

> *"…TB is transmitted by doing heavy jobs like farming and engaging in sexual intercourse with girls and smoking…" [TB Patient, 1 TZ].*
>
> *"…our people have never known what caused TB. That's one thing. They say may be smoke, staying indoor maybe in the kitchen where there is a lot of smoke get you to have TB. Without really knowing the actual causes of TB, so I think our people have very limited knowledge on the cause of TB…*" [Lab In charge, 1 KE].
>
> *" … I first lost appetite, could not eat well, then my health continued to deteriorate, I was told it was malaria, I tried this and that treatment but no improvement...but then I noted that I was sweating a lot at night...they said they had taken my sputum for culture and it took about two months to get the results" [TB Survivor, 1 TZ].*

In contrast to the general community members, people who have suffered from TB and care givers of TB patients had high awareness of TB and basic knowledge of TB disease. The majority of TB patients and their caregivers knew basic facts about TB transmission, common signs and symptoms, and that TB is curable. In addition, some even mentioned about the association between TB and HIV. For example, one interviewee said:

> *"…TB is a chronic illness. TB symptoms are severe cough of more than two weeks, coughing the sputum which are blood mixed, fever during the night and overnight sweating.*'' [TB Survivor, 2 TZ].

Another interviewee mentioned "…*there are two types of TB. Pulmonary TB which affects lungs, a person with this TB coughs severely and this person is infectious but the TB which affects joints, the patient do not cough ad he is not infectious*''.[MDR-TB Patient, 2 TZ]

Despite the high awareness of TB among TB patients and their caregivers, it was noted that most TB patients and caregivers were not aware of TB symptoms before they had suffered from the disease. It was only after diagnosis and getting education from the health care providers that they got to know some basics of TB as one TB survivor commented,

> *"…I really never thought it could be TB because I was coughing and I thought it was a normal cough or something like pneumonia. TB is not a small disease"* [TB Survivor, 3 TZ].

As a result of low awareness of TB disease, recognition and interpretation of TB symptoms and signs among community members, perceived symptom severity was what pushed them as last resort to take their patients to the health facility for diagnosis and treatment, as stated below;

> "…*First the (Tb patient) started coughing and to lost weight, we saw his body changing daily, he lost appetite, he was tired all the time, very much sweating especially at night he was complaining about chest pain. From there his condition continued to be worse, we decided to take him to hospital...*" [*Male Care Giver, 1 KE].*

Another caregiver alluded to this notion by stating

> "…*my son experienced regular fever and coughing for almost four weeks then we brought him to the hospital. But we did not notice its TB". We Tanzanians have a bad habit of taking things easy. We thought it was a normal cough but after four weeks…he was getting very serious, that is when we brought him to the hospital where we found it is TB"*[Female Care Giver, 2 TZ].

Beliefs and use of alternative medicine: Several beliefs about TB disease, its causes and treatment existed in the community that delayed community members to seek care at health facilities. Regarding the cause of TB, some participants believed that it is caused by witchcraft. For example, one TB patient said,

For peer review only - http://bmjopen.bmj.com/site/about/guidelines.xhtml

"...Before I was diagnosed to have this disease, I had consulted more than one traditional healer. My mother believed that, I was bewitched" (TB patient, 3 TZ). In other cases, the participants believed that TB is caused by long uvula as illustrated by one village leader *"...Most of the people know that TB is a disease which affects the throat (KILIMI) [Uvula] and most of them go to traditional healers to remove/cut them thinking that it is the reason why they have severe cough* ''. [Village leader, 1 TZ]

In addition to cultural beliefs about the cause of TB, a variety of beliefs about alternative medicines of TB in the community were widespread as reported by the study participants. The community members did believe that the readily available local medicines in the community have the capacity of curing TB. For example, one TB patient said "*here is a medicine called Irubukoi and most of the people use them to cure TB and pneumonia*'' (TB Patient, 4 TZ]

Apart from the local medicine and traditional healers, some community members believe TB is caused by spirits and can be treated through prayers in churches or mosques as illustrated below:
"...There is this community of Nubis, they are our relatives and we intermarry a lot but they love their things to do with spirits. They believe a sick person is possessed by spirit and hence they go to their spiritual leaders for prayers yet they have TB or HIV..." [TB Care Giver, 3 KE]
"*…We thought he (son) was having spiritual attack and so we took him to the pastor to cast out the spirits…*" (TB care giver, 6 UG)
The beliefs which make people think of other ailments first when they are confronted with TB symptoms and/or make them delay to seek treatment from healthcare facilities or not seek it at all are quite prevalent amongst communities in the region. One healthcare provider shared example of how such beliefs manifest: "*we meet some clients who had non-medical treatment from home, because of the culture. There are people who tried to use herbs. They tried to treat a number of conditions with herbs. And they tend to take too long to seek treatment from healthcare facilities. An attitude like: "this one is culturally known to do it, even if you go for the medical treatment it may not help, let's use this" [Health care provider, 1 KE].*
Another healthcare provider said *"everyone knows about this, even children. Their mothers first buy them syrups over-the-counter not prescribed by the doctor and they try treatment at home. When their treatment fails or seems to be failing that is when they think of going to the clinic. When we ask what treatment they took from home, people often buy first line anti-biotic treatment-someone can buy amoxicillin, if there is no improvement, they change to another antibiotic of different name thinking that there will be improvement thereafter."[Health care provider, 2 TZ]*

Size, taste, and number of pills and side effects of TB treatment: Apart from alternative medicine, beliefs and traditions, the number and size of pills was among the challenges mentioned to interfere with treatment adherence as reported by a male care giver "*...the huge size of these pills sometimes discourages patients from swallowing them...when I used to give the pills to my father he used to complain that they were too big to swallow and thus he wanted the small ones."*[Male care giver, 4 TZ] Another interviewee mentioned *"...the moment you are discovered remember you go, you undergo these 4 tablets plus the other ones 5, plus if you are co-infected more, so its pill burden..."* [TB patient,5 UG]. In a focus group discussion, a female survivor of TB [4 TZ] pointed out bitterness of the pills as another hinderance to taking the medicines and suggested sugar-coating the pills to make them more palatable.

The side effects associated with anti-TB drugs was pointed out as one of the major reasons as to why TB patients apparently fail to complete treatment. Drawing on their lived experience, respondents shared descriptions of the side effects of the drugs *"...All of them (patients on treatment) will complain of dizziness. Others have vomiting if they are not getting used to the medicine. Others reacted to the medicine and lost hearing. What we call neuropathy, the nerves, they will get a lot of pain-you get up in the morning and you are unable to walk. When you step on the ground you feel like you are stepping on thorns and sharp objects."* [Health care provider, 3 TZ].

For peer review only - http://bmjopen.bmj.com/site/about/guidelines.xhtml

Another health care provider added by saying *"…Somebody treated, declared cured, comes back with a cough. About three of them, all of them I managed to send the samples to Mulago for what we call mycology culture. All of them have shown to be having fungal diseases, which is making them weak and when they are coughing this time they don't cough like TB. The cough is severe. They get wasted in a shorter time. That fungus it is not TB. Well the drug you use for treating TB are very difficult drug, in terms of their compromising the immunity. Now, once immunity drop to a certain point and remember the lung is damaged. And still this fungus also takes advantage of the weak immunity and the damaged lung to develop in your body."* [Healthcare provider, 4 UG]

Stigma associated with TB disease: The study found that stigma was rife among the patients, family, relatives, friends and neighbours, including perceived and enacted stigma as well as discrimination. All TB patients interviewed reported experiencing stigma and discrimination from the immediate family members, the community and even at health facilities. The following statements illustrate how TB patients were stigmatized and discriminated

"…My wife separates my bed sheets from theirs, the shoes, utensils, they are kept a little bit separate so that my family and children can`t contract TB'' [Recurrent TB patient- Old man, 6 TZ].

"*…I used to be fearful; ashamed I could not move a lot because I knew people were talking about me. I stayed in the house. I would just get outside next to my house and warm myself in the sun because I did not have visitors, they did not want to come…*" [TB patient, 7 KE].

"*…in other healthcare centres they mistreat the patients by addressing them with inappropriate language, it de-motivates the patients for sure…''* [TB Survivor, 5 KE].

In addition, TB patients are also stigmatized to having HIV too. There was a sense in the community that if one has TB, s/he is most likely to have HIV too. Talking about this misconception, one participant said "*…most of the people know that when a person is having TB then this person is likely to have HIV/AIDS as well…[Community member, 1 TZ]''*. Another one said *"…because they appear emaciated, people might think they have HIV but some may not be having HIV they are just TB without other comorbidity…So I think people should not be afraid the tests… "* [Private practitioner, 1 TZ]

Fear of HIV testing: The policy recommending HIV test for every TB patient together with the perception that TB patient must have HIV, has created fear among people fear to go for TB testing. The community members fear that they will be asked to check for HIV status as well which eventually delays access and use of the TB diagnostic services. The following statements illustrate the fear of HIV testing in the community.

"… people know that if people cough severely it means they have HIV and this makes people fear to go to the hospital for diagnosis and treatment because they think that they will be found to have HIV/AIDS…"[Village leader,2 KE]

"…People fear to go to the hospital for TB diagnosis because they will be diagnosed and if they have TB, HIV will also be tested …" [TB Survivor, 6 TZ]

"…people are scared to go and check for TB because of fear of being tested for HIV. They fear that they will be diagnosed with HIV and TB and when they come back to take medicine, other people will see them at the clinic…" [TB patient, 8 KE].

Socio-economic factors

Costs related to health facilities: A common view amongst most of respondents especially in the rural settings was that costs incurred in using health services including transportation was a significant socio-economic barrier to accessing TB diagnosis and treatment services. For example, one TB patient said "*… when you plan to come to the hospital you need to arrange for bus fair, accommodation cost. You cannot come to town and go back home the same day… It is very far*" [Female TB Patient, 13 TZ]. This view was echoed by another informant who said "*…I use Tz.shs. 2,000/= to pay for the bus transport coming to hospital and returning home…*" [Female Care Giver, 5 TZ].

"…Sputum analysis you pay Ug.shs. 20,000/= for the gram stain that is when you are expecting a bacterial infection: and the ZN (Ziehl-Neelsen) that is now testing for the AFBs (Acid-Fast Bacilli),

For peer review only - http://bmjopen.bmj.com/site/about/guidelines.xhtml

which is for TB, which causes TB. So, the whole test cost Ug.shs.20,000/= Ug.shs. 10,000/= for the gram stain and Ug.shs.10,000/= for the ZN." [TB patient, 9 UG]

"...when you attend hospital you will have to pay for the blood tests which they are so costly and sometime you will be asked to go for a test in private laboratory where tests are more expensive…"[TB survivor , 7 UG]

Despite the costs, geographical accessibility also played a significant role in hindering community members to access to health facilities for TB diagnosis and treatment adherence. One caregiver commented *"...so from here to our hospital is a bit far since we are in the interior and especially when we have rains like this time, to travel from here to the hospital is very hard because accessibility of roads is very hard…"* [Care giver, 6 TZ].

It is the general view of respondents that their respective health care facilities are ill-equipped for TB care, as indicated by shortage of drugs to treat TB, especially Multi-Drug Resistant TB (MDR-TB) and they have to refer patients. A respondent, for example, testified:
"… In case we don't have medicine at that time for MDR-TB you would have to transport the patient to Mulago. The problem is appropriate vehicle to transport them as couldn't travel in the public bus. We must get a pickup (truck) for them to sit and refer them. We usually refer to the national TB centre. But that is not encouraged, because you are now moving around a dangerous disease. It is easier to bring the medicine-source for the medicine and bring it here than sending the person there." [Healthcare provider, 5 UG].
As a result of limited access to health facilities, some patients resort to self-treatment (buying drugs from medical stores) when they get symptoms related to TB that causes delay of diagnosis and treatment. *"...TB patients just go to buy drugs…one says they have pneumonia…they go to a chemist…you just buy cough medication…" [Care Giver, 7KE]*

Lack of food during treatment: Shortage of food was one the socio-economic concerns highlighted for non-adherence to TB medication. This theme recurred throughout the data set as a significant socio-economic barrier to keeping up with medication adherence. Due to lack of food, some patients skipped medication or even stopped medication. For example, one TB patient said
"...there are few times when I skipped my dose if I did not have enough food to eat, because if you take those drugs on an empty stomach, they make you feel very bad for like two three hours". [TB Patient, 10 KE] Another TB patient also echoed this view, "*…TB medicine is a dose of six months and you should take it every day. Others do stop using the medicines because of lack of food*'' [TB patient, 11 TZ].

Work patterns and responsibilities: Working patterns and conditions such as mining not only creates a high-risk environment for TB transmission but also prevented miners from accessing TB diagnosis as well as treatment adherence. For example, one miner commente*d*
"...The young men working in mining areas because we are at a very high risk of contracting TB but when we fall sick, our bosses do not give us anything…"[Community member, 2 TZ]. Apart from working patterns and conditions, family responsibilities among caregivers somehow do play role in hindering caregivers' accessing TB diagnosis services and even keeping up with TB treatment adherence. This notion was shared by one village leader who stated **"***...most of the TB patients are poor, so when they feel relief even in the middle of the dose, they tend to stop and get back to work in order to earn a living…"* [Village leader, 3 TZ**].**

Lifestyle: The nomadic lifestyle among the Maasai tribe also poses as a socioeconomic barrier to seeking TB diagnosis and treatments services at health facilities. Moving from one place to another greatly interferes with medication adherence. *"...Maasai are living very far from the health centres because they tend to move from place to place far from town looking for the pasture for their animals…*"[TB Care Giver,8 TZ]. Alcohol consumption among the young men in the societies have also shown to be a contributing factor to non-adherence with TB medications. For example, one miner

For peer review only - http://bmjopen.bmj.com/site/about/guidelines.xhtml

commented "*…most of the young men in this street are alcohol drinkers and this tendency disturbs the adherence to TB medicines…*'' [Community member, Miner, 3 TZ].

Discussion

The study aimed to describe the socioeconomic and cultural factors negatively affecting the uptake and utilisation of diagnostic and treatment tools for tuberculosis in East Africa. The findings represent the views of various key actors including patients, survivors, caregivers, healthcare workers and administrators, and policymakers. The socioeconomic and cultural issues, knowledge of disease signs and causes, perverted beliefs and use of alternative medicine, stigma and deterrent costs of care identified by this study are fundamental and can be generalized to other disease conditions. For instance, anecdotal information shows COVID-19 has caused rise in stigmatization of cough and use of alternative medicines. The recently reported decline in TB notification could be reflecting people fearing to seek formal treatment for cough and resorting to purchasing medicines over-the-counter or using traditional herbs[29]

Lack of knowledge or correct information about TB transmission and treatment was among the prominent factors hindering timely and successful diagnosis and treatment of tuberculosis. These findings concur with what others studies have found on this topic[14,15,20,21,30,31]. Shortage of communication channels like radios, TVs, and internet exacerbate lack of awareness of TB and existing medical remedies among people mostly in rural areas and mining industry. Poor or no electricity supply complicate the use of TVs and computers. Misinformation which is currently rampant in the COVID-19 era has always been a hinderance to uptake medical interventions, and can only be counteracted by proactive sharing of facts.

Access and use of health technologies is further compromised by poor transport infrastructure, high illiteracy rate, and lack of well-equipped health care facilities and specialized health care providers[32]. Analysis of the health system barriers (first part of this study) points to the centralized implementation model as the most cost-effective of ensuring maximal uptake and utilisation of high-tech diagnostic technologies. However, this can only be effective if the governments invested in good transport infrastructure connecting rural and urban areas to ensure timely sample referral and delivery of diagnostic samples. Investing in information communication technologies would not only ensure higher awareness of TB and its care services but also communicating laboratory results to patients once tests are completed. Hiring and retaining well trained healthcare practitioners and giving them refresher training on novel health technologies cannot be more emphasized.

The current tuberculosis treatment regimen is a combination of four antibiotics for six months in drug sensitive TB and more months for drug resistant TB[33]. Presence of comorbidity like HIV is worsens the pill burden. The pill burden (size and number of pills) was highlighted by participants as major hinderance to adherence to treatment. Side effects such as vomiting and loss of hearing worsened the situation. This calls for medicines developers to find innovative ways of combining drugs into single pills and less bitter to increase palatability. Finding fewer toxic drugs or combinations of drugs should be the main agenda of drug manufacturers and clinical trials. Increasing patient – practitioner communication and interaction has been suggested as one of the ways to manage and mitigate side effects caused by medicines and ensuring treatment adherences[34]. Patients should be well informed about the side effects, pill burden as well as supportive nutritional foods they need while on treatment. Our study found stigma to TB infection was mostly associated with HIV infection, since the general community perception is that anyone testing positive for TB is also HIV infected. As much as the perception is incorrect because HIV-TB coinfection is estimated at 40% in East Africa, it shapes the attitudes in the negative direction[35]. This contrasts the TB stigma in West Africa where TB is associated with ethnic or family curse, being grossly unhygienic and very contagious so much that even healthcare practitioners shun working with TB patients[36]. Consequently, stigma make people more inclined to keeping their ailment a secret and reduces willingness to seek medical care. This in turn stops or delays access to appropriate diagnostic and treatment services[18,19]. We believe poor understanding of the causes and signs of disease like TB creates a knowledge gap that people tend to fill with superstition and stigma. Those purposed to have knowledge such as healthcare practitioners, their stigma may be driven by lack of personal protective equipment (PPE) for which they ensure safety by avoiding patients with contagious diseases like TB. Sensitisation of both community and healthcare

For peer review only - http://bmjopen.bmj.com/site/about/guidelines.xhtml

practitioners coupled with provision of appropriate PPE would go a long way in resolving the stigma issue.

Beliefs and myths about the causes and transmission for TB including being related to witchcraft, family curse and having a long Uvula ***(known as Kilimi in Swahili both in TZ and KE)*** as cause of recurrent coughing raised stigma and discrimination of TB patients. As a result, the patient pathway to care is complicated as most patients resort to seeking remedy from traditional healers, palatine uvula cutters before going to the formal healthcare centres. These kinds of behaviours and beliefs have been reported in other African countries[37]. Action follows belief and so someone who believes witchcraft is the cause of TB will by default consult a traditional healer first, which prolongs the pathway to diagnosis and treatment at formal healthcare facilities[34].

One of the greatest challenges during diagnosis and treatment process revealed in our study is the transportation costs and other indirect costs such as food incurred by families living in rural areas during their journey of accessing TB care from diagnosis and throughout treatment follow up. As a result of these costs compounded by long distance to healthcare facilities, some families delay to seek TB diagnosis and treatment in health facilities. Lack of food makes adhering to medication an uphill task as some of the medicines are toxic when taken on empty stomach. The theme of debilitating costs of TB care resonates with findings by many studies elsewhere and deserves urgent attention[14,32,34,38–40]. Efforts to maximise uptake and utilisation of health technologies can only be effective if poverty, the main confounder of the poverty related diseases like TB is addressed.

Drawing from our findings we believe the largest "road block" to uptake and utilisation of diagnostic and treatment tools occurs at the primary point of access in the patient pathway. This is the point between feeling poorly and getting diagnosis. It there makes more sense for most investment to be made to shorten the path to access of healthcare services which in turn would increase uptake and utilisation of health technologies (Fig. 2)

Conclusion and Recommendations

Our study has shown that socioeconomic and cultural factors are important barriers to uptake and utilisation of diagnostic and treatment tools. While our study was done in the context of tuberculosis, we believe these challenges are fundamental and can apply to any other disease. Resolving awareness/knowledge gaps, beliefs, stigma and improving socioeconomic welfare of affected communities is an antidote that cuts across many disease conditions. We recommend that equal weight be given to resolving both health system and socioeconomic – cultural barriers in order to maximise uptake and utilisation of health technologies by communities who most need them. Increasing sensitization of healthcare practitioners and service users is the first cost-effective step to maximise uptake of health research innovations into practice. Learning from end users by diagnostics and medicines developers will enable them develop effective diagnostic and treatment tools. Social protection programmes to people working in high risk industries like mining and interventions to reduce use of dirty sources of energy like firewood, charcoal, coal etc are critical for reducing the burden of respiratory diseases like TB. Focusing investment at the primary point of healthcare access is critical to maximising uptake and utilisation of diagnostic and treatment tools.

Data sharing statement

Extra is available upon request and meeting the confidentiality and use of data requirements signed between authors and participants. Email ws31@st-andrews.ac.uk to register your request.

Acknowledgements

The authors acknowledge the contributions of Dr Ewan Chirnside and Prof Gibson Kibiki in the initial study conception, design and implementation of the study. Prof Kibiki also played a big role in linking the TWENDE consortium to East African Health Research Commission (EAHRC). As a result of his effort, EAHRC officially adopted TWENDE as a research consortium for generating evidence needed for translation of health technologies to policy and practice in the East African Community. Special memories of Dr Alphonse Okwera who was the Uganda country PI for the TWENDE study and sadly

For peer review only - http://bmjopen.bmj.com/site/about/guidelines.xhtml

passed away before this manuscript was written. Thanks to Ms Norah Owaraga who coordinated the training of qualitative researchers in the TWENDE consortium. We also acknowledge Dr Veronica O'Carroll and Dr Stella-Neema for qualitative expertise they to proof read the manuscript that improved the cogency of the manuscript.

Funding

The study was funded by the European and Developing Countries Clinical Trials Partnership (EDCTP), grant TWENDE-EDCTP-CSA-2014-283.

Author contributions

Study design: WS, SHG, NET, EA, BTM, MJ; Data collection and collation: EFM, FO, ES, BM, SM, HM, KK, AL, IM; Drawings: AA & WS; Data analysis: EFM, FO, ES, HM; Manuscript drafting: EFM, ES, FO, WS; Manuscript review: All co-authors.

References

1 World Health Organization. Are Updated Every Year . for the Tuberculosis. 2019. https://www.who.int/tb/publications/global_report/en/

2 WHO. Global TB Report. Geneva 27, Switzerland: 2014. ISBN 978 924 1564809

3 Murray CJL, Ortblad KF, Guinovart C, *et al.* Global , regional , and national incidence and mortality for HIV , tuberculosis , and malaria during 1990 – 2013 : a systematic analysis for the Global Burden of Disease Study 2013. 2014;**6736**:1–66. doi:10.1016/S0140-6736(14)60844-8

4 Lönnroth K, Castro KG, Chakaya JM, *et al.* Tuberculosis 1 Tuberculosis control and elimination 2010 – 50 : cure , care , and social development. *Lancet* 2015;**375**:1814–29. doi:10.1016/S0140-6736(10)60483-7

5 Zumla A, Gant V, Bates M, *et al.* Rapid diagnostics urgently needed for killer infections. *Lancet Respir Med* 2013;**1**:284–5. doi:10.1016/S2213-2600(13)70099-7

6 Lawn SD, Mwaba P, Bates M, *et al.* Advances in tuberculosis diagnostics: The Xpert MTB/RIF assay and future prospects for a point-of-care test. *Lancet Infect Dis* 2013;**13**:349–61. doi:10.1016/S1473-3099(13)70008-2

7 Parsons LM, Somoskövi Á, Gutierrez C, *et al.* Laboratory diagnosis of tuberculosis in resource-poor Countries: Challenges and opportunities. *Clin Microbiol Rev* 2011;**24**:314–50. doi:10.1128/CMR.00059-10

8

9 Pandey V, Singh P, Singh S, *et al.* SeeTB : A novel alternative to sputum smear microscopy to diagnose tuberculosis in high burden countries. 2019;:1–10. doi:10.1038/s41598-019-52739-9

10 Zijenah LS, Zijenah S. The World Health Organization Recommended TB Diagnostic Tools Organization Recommended TB The World Health Diagnostic Tools. doi:10.5772/intechopen.73070

11 Boehme CC, Nicol MP, Nabeta P, *et al.* Feasibility, diagnostic accuracy, and effectiveness of decentralised use of the Xpert MTB/RIF test for diagnosis of tuberculosis and multidrug resistance: A multicentre implementation study. *Lancet* 2011;**377**:1495–505. doi:10.1016/S0140-6736(11)60438-8

12 Nahid P, Pai M, Hopewell PC. Advances in the diagnosis and treatment of tuberculosis. *Proc Am Thorac Soc* 2006;**3**:103–10. doi:10.1513/pats.200511-119JH

13 Albert H, Bwanga F, Mukkada S, *et al.* Rapid screening of MDR-TB using molecular Line Probe Assay is feasible in Uganda. *BMC Infect Dis* 2010;**10**. doi:10.1186/1471-2334-10-41

14 Vries SG De, Cremers AL, Heuvelings CC, *et al.* Series Tuberculosis in hard-to-reach populations 1 Barriers and facilitators to the uptake of tuberculosis diagnostic and treatment services by hard-to-reach populations in countries of low and medium tuberculosis incidence : a systematic review of qualit. 2017;**3099**:1–16. doi:10.1016/S1473-3099(16)30531-X

15

16 Watermeyer J, Penn C. Community perspectives on tuberculosis care in rural South Africa.

For peer review only

For peer review only - http://bmjopen.bmj.com/site/about/guidelines.xhtml

2018;:1–9. doi:10.1111/hsc.12637
17 Ngamvithayapong J, Winkvist A, Diwan V. High AIDS awareness may cause tuberculosis patient delay : results from an HIV epidemic area , Thailand. 2000;:1413–9.
18 Courtwright A. Tuberculosis and Stigmatization : Pathways and Interventions. ;**125**:34–42.
19 Jittimanee SX, Nateniyom S, Kittikraisak W, *et al.* Social Stigma and Knowledge of Tuberculosis and HIV among Patients with Both Diseases in Thailand. 2009;**4**:6–12. doi:10.1371/journal.pone.0006360
20 Kipp AM, Pungrassami P, Stewart PW, *et al.* Study of tuberculosis and AIDS stigma as barriers to tuberculosis treatment adherence using validated stigma scales. 2011;**15**:1540–5.
21 Royce Rachel A. Americans in Three Southeastern Counties in the United States : 2017;:1–21. doi:10.1007/s40615-015-0200-1.Tuberculosis
22 Gelaw SM. Socioeconomic Factors Associated with Knowledge on Tuberculosis among Adults in Ethiopia. 2016;**2016**.
23 Stuckler D, Basu S, Mckee M, *et al.* Mining and Risk of Tuberculosis in Sub-Saharan Africa. 2011;**101**:524–30. doi:10.2105/AJPH.2009.175646
24 Crush J, Williams B, Gouws E, *et al.* Migration and HIV / AIDS in South Africa. 2005;**22**.
25 Graneheim UH, Lundman B. Qualitative content analysis in nursing research: Concepts, procedures and measures to achieve trustworthiness. *Nurse Educ Today* 2004;**24**:105–12. doi:10.1016/j.nedt.2003.10.001
26 Piergiorgio Corbetta. *Social Research : Theory , Methods and Techniques The Use of Documents*. London: : Sage Publications 2003. doi:http://dx.doi.org/10.4135/9781849209922.n11
27 Barbour RS. Checklists for improving rigour in qualitative research: a case of the tail wagging the dog? *Bmj* 2001;**322**:1115–7. doi:10.1136/bmj.322.7294.1115
28 2007 - Creswell - Data Analysis and Representation.pdf.
29 WHO 2020. Infection Prevention and Control guidance for Long-Term Care Facilities in the context of COVID-19. *Interim Guid World Heal Organ* 2020;:1–5.
30 Kigozi NG, Heunis JC, Engelbrecht MC, *et al.* Tuberculosis knowledge , attitudes and practices of patients at primary health care facilities in a South African metropolitan : research towards improved health education. 2017;:1–8. doi:10.1186/s12889-017-4825-3
31 Asres M, Gedefaw M, Kahsay A, *et al.* Patients' delay in seeking health care for tuberculosis diagnosis in east gojjam zone, northwest Ethiopia. *Am J Trop Med Hyg* 2017;**96**:1071–5. doi:10.4269/ajtmh.16-0892
32 Ereso BM, Yimer SA, Gradmann C, *et al.* Barriers for tuberculosis case finding in Southwest Ethiopia: A qualitative study. *PLoS One* 2020;**15**:1–20. doi:10.1371/journal.pone.0226307
33 WHO 2017. *Treatment of tuberculosis.* Switzerland: 1954. ISBN 978-92-4-155000-0%0A©
34 Boru CG, Shimels T, Bilal AI. Factors contributing to non-adherence with treatment among TB patients in Sodo Woreda, Gurage Zone, Southern Ethiopia: A qualitative study. *J Infect Public Health* Published Online First: 2017. doi:10.1016/j.jiph.2016.11.018
35 Bonnington O, Wamoyi J, Ddaaki W, *et al.* Changing forms of HIV-related stigma along the HIV care and treatment continuum in sub-Saharan Africa : a temporal analysis. 2017;:1–6. doi:10.1136/sextrans-2016-052975
36 Dodor EA, Kelly SJ. Manifestations of tuberculosis stigma within the healthcare system: The case of Sekondi-Takoradi Metropolitan district in Ghana. *Health Policy (New York)* 2010;**98**:195–202. doi:10.1016/j.healthpol.2010.06.017
37 Amo-adjei J, Kumi-kyereme A. Myths and misconceptions about tuberculosis transmission in Ghana. 2013.
38 Woimo TT, Yimer WK, Bati T, *et al.* The prevalence and factors associated for anti-tuberculosis treatment non-adherence among pulmonary tuberculosis patients in public health care facilities in South Ethiopia : a cross-sectional study. 2017;:1–10. doi:10.1186/s12889-017-4188-9
39 Otwombe KN, Variava E, Holmes CB, *et al.* Predictors of delay in the diagnosis and treatment of suspected tuberculosis in HIV co-infected patients in South Africa. *Int J Tuberc Lung Dis* 2013;**17**:1199–205. doi:10.5588/ijtld.12.0891
40 Tadesse T, Demissie M, Berhane Y, *et al.* Long distance travelling and financial burdens discourage tuberculosis DOTs treatment initiation and compliance in Ethiopia : a qualitative

For peer review only - http://bmjopen.bmj.com/site/about/guidelines.xhtml

study. 2013.

Figure legends

Figure 1: The map showing the surveyed regions and counties in three East African Community Countries (EAC).

Figure 2: Diagrammatic representation of the impediments of access, uptake and utilisation of diagnostic and treatment tools in the patient pathway. Most hinderance is experienced at the primary level where the patient first seeks healthcare intervention during their ailment. The path is not straight reflecting the different barriers and routes the patient goes through to get- or adhere to- care.

For peer review only

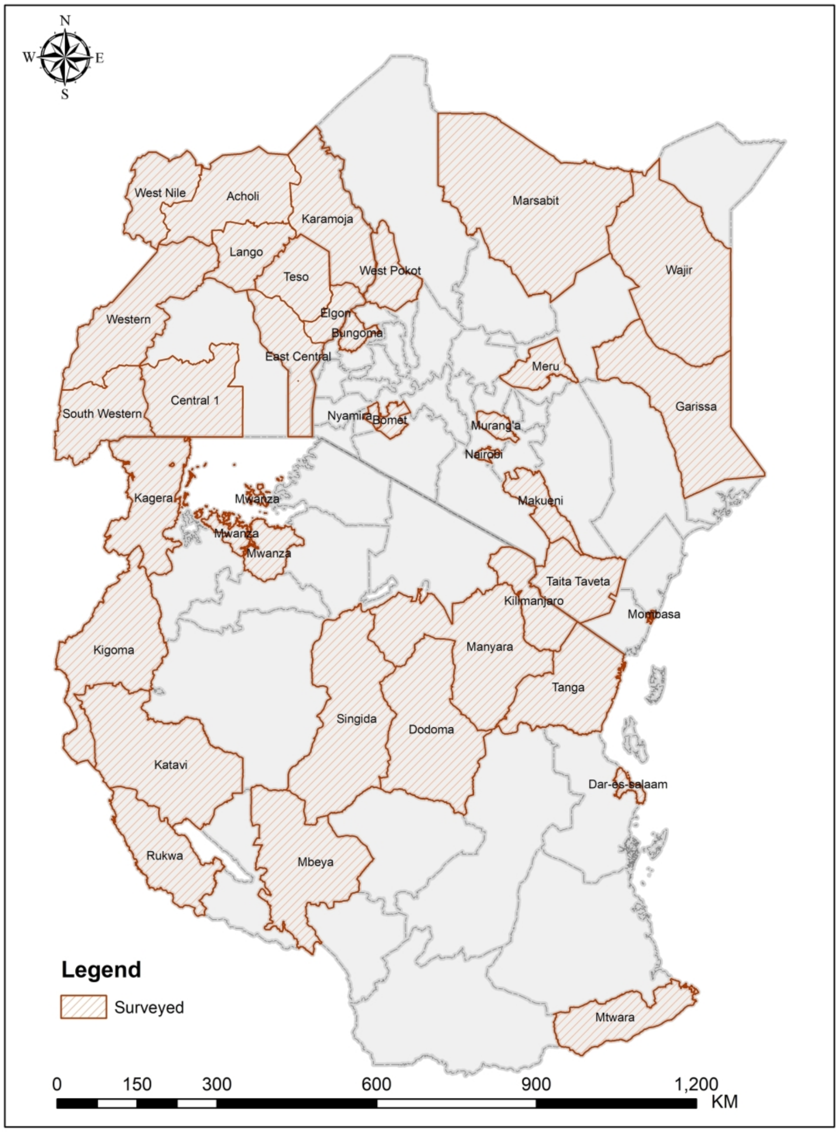

Figure 1: The map showing the surveyed regions and counties in three East African Community Countries (EAC).

215x279mm (300 x 300 DPI)

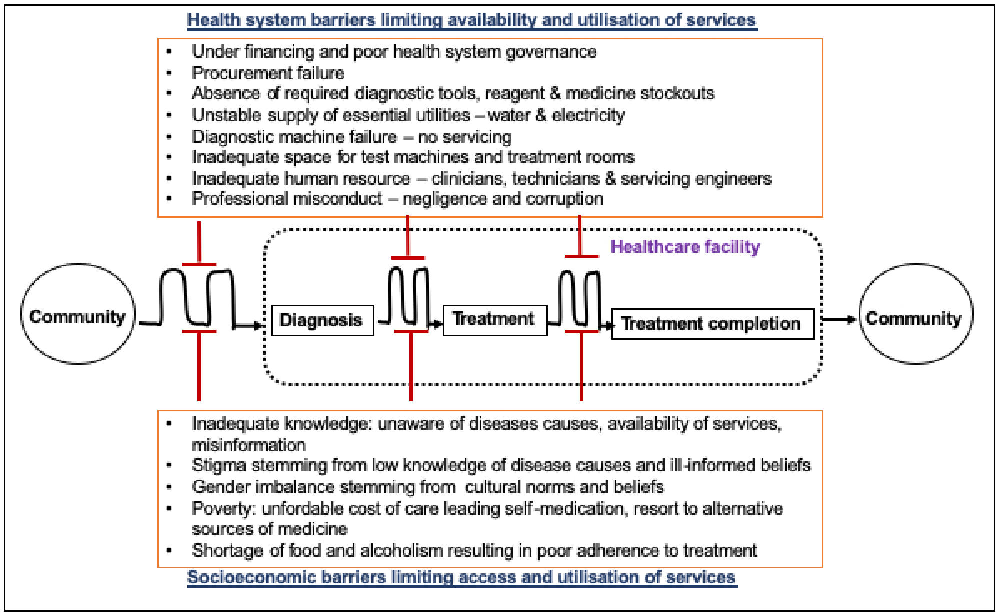

Figure 2: Diagrammatic representation of the impediments of access, uptake and utilisation of diagnostic and treatment tools in the patient pathway. Most hinderance is experienced at the primary level where the patient first seeks healthcare intervention during their ailment. The path is not straight reflecting the different barriers and routes the patient goes through to get- or adhere to- care.

281x174mm (54 x 54 DPI)

Revised Standards for Quality Improvement Reporting Excellence (SQUIRE 2.0)
September 15, 2015

Text Section and Item Name	Section or Item Description
Notes to authors	• The SQUIRE guidelines provide a framework for reporting new knowledge about how to improve healthcare<br>• The SQUIRE guidelines are intended for reports that describe system level work to improve the quality, safety, and value of healthcare, and used methods to establish that observed outcomes were due to the intervention(s).<br>• A range of approaches exists for improving healthcare. SQUIRE may be adapted for reporting any of these.<br>• Authors should consider every SQUIRE item, but it may be inappropriate or unnecessary to include every SQUIRE element in a particular manuscript.<br>• The SQUIRE Glossary contains definitions of many of the key words in SQUIRE.<br>• The Explanation and Elaboration document provides specific examples of well-written SQUIRE items, and an in-depth explanation of each item.<br>• Please cite SQUIRE when it is used to write a manuscript.
Title and Abstract	
1. Title	Found on page 1: Our tittle encapsulates the issue under investigation, how it was investigated and the need to address it for improved healthcare provision.
2. Abstract	Found on page 2: It gives brief background and objective of the study, setting, participants and approaches, results and conclusion.
Introduction	Why did you start?
3. Problem Description	Found on Page 3 giving background summary of the problem.
4. Available knowledge	Found on Page 2 first paragraph of the summary

5. Rationale	Found on Page 2 and 3 as part of the summary giving reasons for the study, the strength and limitations.
6. Specific aims	Found on page 2 and 3 as part of the abstract and summary.
Methods	What did you do?
7. Context	Found on page 3 1st paragraph of the methods
8. Intervention(s)	Found on page 4 – gives details of the methods used
9. Study of the Intervention(s)	Found on page 3 and 4 describing the qualitative approach used in the investigations.
10. Measures	Found on page 4 under data management and analysis.
11. Analysis	Found on page 4 – data management and analysis.
12. Ethical Considerations	Found on page 4 under ethical approval and consent to participate.
Results	What did you find?
13. Results	Found on page 5 giving a qualitative description of the findings.
Discussion	What does it mean?
14. Summary	Found on page 2

For peer review only

15. Interpretation	Found on page 10 and 11 as part of the discussion and conclusion.
16. Limitations	Found on 2-3 as part of the summary.
17. Conclusions	Found on page 10 in the last paragraph of the discussion with diagrammatic illustration, conclusion and recommendations.
Other information	
18. Funding	Found on page 12.

For peer review only

Table 2. Glossary of key terms used in SQUIRE 2.0. This Glossary provides the intended meaning of selected words and phrases as they are used in the SQUIRE 2.0 Guidelines. They may, and often do, have different meanings in other disciplines, situations, and settings .

Assumptions
Reasons for choosing the activities and tools used to bring about changes in healthcare services at the system level.

Context
Physical and sociocultural makeup of the local environment (for example, external environmental factors, organizational dynamics, collaboration, resources, leadership, and the like), and the interpretation of these factors ("sense-making") by the healthcare delivery professionals, patients, and caregivers that can affect the effectiveness and generalizability of intervention(s).

Ethical aspects
The value of system-level initiatives relative to their potential for harm, burden, and cost to the stakeholders. Potential harms particularly associated with efforts to improve the quality, safety, and value of healthcare services include opportunity costs, invasion of privacy, and staff distress resulting from disclosure of poor performance.

Generalizability
The likelihood that the intervention(s) in a particular report would produce similar results in other settings, situations, or environments (also referred to as external validity).

Healthcare improvement
Any systematic effort intended to raise the quality, safety, and value of healthcare services, usually done at the system level. We encourage the use of this phrase rather than "quality improvement," which often refers to more narrowly defined approaches.

Inferences
The meaning of findings or data, as interpreted by the stakeholders in healthcare services - improvers, healthcare delivery professionals, and/or patients and families

Initiative
A broad term that can refer to organization-wide programs, narrowly focused projects, or the details of specific interventions (for example, planning, execution, and assessment)

Internal validity
Demonstrable, credible evidence for efficacy (meaningful impact or change) resulting from introduction of a specific intervention into a particular healthcare system.

Intervention(s)
The specific activities and tools introduced into a healthcare system with the aim of changing its performance for the better. Complete description of an intervention includes its inputs, internal activities, and outputs (in the form of a logic model, for example), and the mechanism(s) by which these components are expected to produce changes in a system's performance.

Opportunity costs

For peer review only

Loss of the ability to perform other tasks or meet other responsibilities resulting from the diversion of resources needed to introduce, test, or sustain a particular improvement initiative

Problem

Meaningful disruption, failure, inadequacy, distress, confusion or other dysfunction in a healthcare service delivery system that adversely affects patients, staff, or the system as a whole, or that prevents care from reaching its full potential

Process

The routines and other activities through which healthcare services are delivered

Rationale

Explanation of why particular intervention(s) were chosen and why it was expected to work, be sustainable, and be replicable elsewhere.

Systems

The interrelated structures, people, processes, and activities that together create healthcare services for and with individual patients and populations. For example, systems exist from the personal self-care system of a patient, to the individual provider-patient dyad system, to the microsystem, to the macrosystem, and all the way to the market/social/insurance system. These levels are nested within each other.

Theory or theories

Any "reason-giving" account that asserts causal relationships between variables (causal theory) or that makes sense of an otherwise obscure process or situation (explanatory theory). Theories come in many forms, and serve different purposes in the phases of improvement work. It is important to be explicit and well-founded about any informal and formal theory (or theories) that are used.

For peer review only

BMJ Open

Qualitative assessment of the impact of socioeconomic and cultural barriers on uptake and utilisation of tuberculosis diagnostic and treatment tools in East Africa: A cross-sectional study

Journal:	*BMJ Open*
Manuscript ID	bmjopen-2021-050911.R1
Article Type:	Original research
Date Submitted by the Author:	19-May-2021
Complete List of Authors:	Msoka, Elizabeth ; Kilimanjaro Christian Medical University College, Clinical Trial<br>Orina, Fred; Kenya Medical Research Institute<br>Sanga, Erica; NIMR-Mbeya Medical Research Programme, Social Science<br>Miheso, Barbara; Kenya Medical Research Institute<br>Mwanyonga, Simeon; NIMR-Mbeya Medical Research Programme<br>Meme, Helen; Kenya Medical Research Institute<br>Kiula, Kiula; Kilimanjaro Christian Medical Centre; The University of Dodoma<br>Liyoyo, Alphonce; Kilimanjaro Christian Medical Centre<br>Mwebaza, Ivan; Makerere University<br>Aturinde, Augustus; Lund University, Department of Physical Geography and Ecosystem Science; Kyambogo University, Department of Physical Geography and Ecosystem Science<br>Joloba, Moses; Makerere University College of Health Sciences<br>mmbaga, blandina; Kilimanjaro Christian Medical University College, ; Kilimanjaro Clinical Researxh Institute<br>Amukoye, Evans; Kenya Medical Research Institute<br>Ntinginya, Nyanda; NIMR-Mbeya Medical Research Programme<br>Gillespie, Stephen; University of St Andrews<br>Sabiiti, Wilber; University of St Andrews, School of Medicine
**Primary Subject Heading**:	Health services research
Secondary Subject Heading:	Diagnostics, Health policy, Infectious diseases, Qualitative research
Keywords:	Diagnostic microbiology < INFECTIOUS DISEASES, Molecular diagnostics < INFECTIOUS DISEASES, Health policy < HEALTH SERVICES ADMINISTRATION & MANAGEMENT, Tuberculosis < INFECTIOUS DISEASES, Public health < INFECTIOUS DISEASES

SCHOLARONE™
Manuscripts

I, the Submitting Author has the right to grant and does grant on behalf of all authors of the Work (as defined in the below author licence), an exclusive licence and/or a non-exclusive licence for contributions from authors who are: i) UK Crown employees; ii) where BMJ has agreed a CC-BY licence shall apply, and/or iii) in accordance with the terms applicable for US Federal Government officers or employees acting as part of their official duties; on a worldwide, perpetual, irrevocable, royalty-free basis to BMJ Publishing Group Ltd ("BMJ") its licensees and where the relevant Journal is co-owned by BMJ to the co-owners of the Journal, to publish the Work in this journal and any other BMJ products and to exploit all rights, as set out in our licence.

The Submitting Author accepts and understands that any supply made under these terms is made by BMJ to the Submitting Author unless you are acting as an employee on behalf of your employer or a postgraduate student of an affiliated institution which is paying any applicable article publishing charge ("APC") for Open Access articles. Where the Submitting Author wishes to make the Work available on an Open Access basis (and intends to pay the relevant APC), the terms of reuse of such Open Access shall be governed by a Creative Commons licence – details of these licences and which Creative Commons licence will apply to this Work are set out in our licence referred to above.

Other than as permitted in any relevant BMJ Author's Self Archiving Policies, I confirm this Work has not been accepted for publication elsewhere, is not being considered for publication elsewhere and does not duplicate material already published. I confirm all authors consent to publication of this Work and authorise the granting of this licence.

For peer review only

Qualitative assessment of the impact of socioeconomic and cultural barriers on uptake and utilisation of tuberculosis diagnostic and treatment tools in East Africa: A cross-sectional study

Elizabeth F. Msoka[1,] MSc, Fred Orina[2] MSc, Erica Sanga[3]PhD, Barbara Miheso[2]BSc, Simeon Mwanyonga[4]BA, Helen Meme[2]MD, Kiula Kiula[5]MA, Alphonce Liyoyo[,6]MPH, Ivan Mwebaza[7]MSc, Augustus Aturinde[8,9]PhD, Moses Joloba[7]PhD, Blandina Theophil Mmbaga[1]PhD, Evans Amukoye[2]MD, Nyanda Elias Ntinginya[4] PhD, Stephen H Gillespie[10]DSc, Wilber Sabiiti[10]PhD

Author Affiliations

1. Kilimanjaro Christian Medical Centre, Moshi, Tanzania
2. Kenya Medical Research Institute (KEMRI), Nairobi, Kenya.
3. National Institute of Medical Research-Mwanza Medical Research Centre, Mwanza, Tanzania
4. National Institute of Medical Research - Mbeya Medical Research Centre (NIMR-MMRC), Mbeya, Tanzania.
5. Institute of Development studies, University of Dodoma, Dodoma, Tanzania.
6. Kibong'oto Infectious Diseases Hospital, Mae Street, Lomakaa road, Sanya Juu, Siha Kilimanjaro, Tanzania
7. School of Biomedical Sciences, College of Health Sciences, Makerere University, Kampala, Uganda.
8. Department of Physical Geography and Ecosystem Science, Lund University, Sweden.
9. Department of Lands and Architectural Studies, Kyambogo University, Kampala, Uganda.
10. Division of Infection and Global Health, School of Medicine, University of St Andrews, St Andrews, United Kingdom.

Corresponding Author:

Elizabeth Francis Msoka
Kilimanjaro Clinical Research Institute
P.O BOX 2236
Kilimanjaro-Moshi, Tanzania
Email: e.fbright@kcri.ac.tz
Tel +255(27)2754201

Abstract

For peer review only

For peer review only - http://bmjopen.bmj.com/site/about/guidelines.xhtml

Objectives: Early diagnosis and timely treatment are key elements of a successful healthcare system. We assessed the role of socioeconomic and cultural norms in accelerating or decelerating uptake and utilisation of health technologies into policy and practice.

Setting: Secondary and tertiary level healthcare facilities in three East African countries. Level of healthcare facility (HCF) was selected based on the WHO recommendation for implantation of tuberculosis (TB) molecular diagnostics.

Participants: Using implementation of TB diagnostics as a model, we purposively selected participants (TB patients, carers, survivors, healthcare practitioners, community members, opinion leaders and policymakers) based on their role as stakeholders. In-Depth Interviews (IDIs), Key Informant Interviews and Focus Group Discussions (FGDs) were held to collect the data between 2016-18. The data were transcribed, translated, coded and analysed by thematic-content analysis.

Results: A total of 712 individuals participated in the study. Socioeconomic and cultural factors such as poverty, stigma, and inadequate knowledge about causes of disease and available remedies, cultural beliefs were associated with low access and utilisation of diagnostic and treatment tools for tuberculosis. Poverty made people hesitate to seek formal healthcare resulting in delayed diagnosis and resorting to self-medication and cheap herbal alternatives. Fear of stigma made people hide their sickness and avoid reporting for follow-up treatment visits. Inadequate knowledge and beliefs were fertile ground for aggravated stigma and believing that diseases like TB are caused by spirits and thus cured by spiritual rituals or religious prayers. Cultural norms were also the basis of gender-based imbalance in accessing care, "I could not go to hospital without my husband's permission", TB survivor.

Conclusion: Our findings show that socioeconomic and cultural factors are substantial "roadblocks" to accelerating the uptake and utilisation of diagnostic and treatment tools. Resolving these barriers should be given equal attention as is to health system barriers.

Keywords: TB, Maximizing uptake, utilisation of diagnostic, treatment tools, police and practice, impact, socio economic, cultural norms, East Africa

Strengths and limitations

- This is a qualitative assessment using triangulated data sources to strengthen the validity of results.
- Valuable insights into the role of socioeconomic and cultural barriers on access and utilization of tuberculosis technologies.
- Investigators were experienced and well-trained on qualitative data collection.
- Views of 712 participants may not necessarily represent views of everyone in East Africa.
- Participant's ability and/or inability to recall past events may limit the accuracy of the data collected.

For peer review only

For peer review only - http://bmjopen.bmj.com/site/about/guidelines.xhtml

Introduction

Tuberculosis (TB) claims more than a million lives every year of which the poorest of the world are most affected[1–4]. Considerable technological advances have been made in the diagnosis and treatment of TB but they remain less accessible to the communities that need it the most[5–7]. Only two thirds of the global reported TB cases are confirmed by a laboratory test[2]

Tanzania, Uganda and Kenya are among the 30 countries with highest burden of tuberculosis as well as TB/HIV co-infection in the world[1]. The high tuberculosis burden is characterised by poor diagnostic capability where many of high burdened countries still depend on microscopy, a less sensitive tool for diagnosis[8–11]. Each of the three countries has a national TB and Leprosy control programme (NTLP) through which TB services are provided[12]. Diagnosis and treatment are free though the pre-diagnosis screening that most patients go through is not free. The length of time taken to getting proper diagnosis depends on the healthcare worker's ability to recognise TB symptoms and person's health seeking behaviour. Although medicines are free, patient care and transports costs to and from healthcare facilities. WHO approved Molecular diagnostics, Xpert MTB/RIF and Line probe assay for rapid detection of tuberculosis and drug resistance that have been found to be implementable though the socioeconomic changes associated with their uptake and utilisation are less elucidated[10,13].

Knowledge of disease, social and cultural issues have been highlighted as important factors in the patient diagnosis and treatment pathway of TB[14]. Studies conducted elsewhere regarding TB and social cultural barriers have revealed that there is a complex interplay between contextual factors and community understanding of the disease. Cultural beliefs about causality and treatment-seeking paths were often mentioned in conjunction with biomedical views[15]. There was a strong interface between TB and HIV in communities, and knowledge of TB is often confused with HIV, additionally HIV-related stigma has been extended to those living with TB[15]. The stigma of TB infection being related to HIV infection has been shown to have an impact on patients seeking treatment and their adherence to treatment[16–18]. These cultural barriers have been reported in most African communities where TB infection is associated or linked to witchcraft as responsible for leading to delay in accessing diagnosis and treatment of TB[19–21]. Interestingly, lack of access to media was highlighted as significantly associated with low tuberculosis knowledge[22].

Socioeconomic activities such as mining are reported high risk for communicable illness including tuberculosis and HIV/AIDS[19]. Population migration has been shown to facilitate transmission of TB as well as causing treatment disruptions [23] [24]. We set out to investigate these factors and the ways they impede uptake of health technologies in order to inform models for resolution in the East African region and the low- and medium- income countries setting at large. We show that socioeconomic and cultural norms substantially limit access and utilisation to healthcare services and resolving them is fundamental to having well-functioning health systems.

Methods

Approach

This was a qualitative part of a larger mixed methods study assessing health system and wider community impediments and opportunities to unlock them in order to achieve effective uptake and utilisation of diagnostic and treatment tools. Implementation of WHO approved tuberculosis diagnostics was used as benchmark to evaluate the phenomenon. The study was conducted between Jan and Dec 2017 in Uganda, Kenya and Tanzania. At the time of the study, only Kenya was defined as a middle-income country, and it has now been joined by Tanzania in this classification. This means the Tanzania results in this study reflect a low-income country setting.

For peer review only - http://bmjopen.bmj.com/site/about/guidelines.xhtml

Study design, participants and researchers

This was a cross-sectional study targeting TB- patients and survivors, care givers, general members of the community served by the healthcare facility (HCF), healthcare practitioners, opinion leaders, local government authorities and policy/decision makers at local and national levels from these three countries. An interdisciplinary team of researchers consisting of social scientists, biomedical scientists and clinicians conducted the study.

Context

Although the study countries: Kenya, Tanzania and Uganda are high TB burden countries, the study did not aim to study TB as a disease but as a prism to look into the challenges to increasing access to health technologies. Although there are pockets of uniqueness in each country, there is substantial level of cross border interaction and similarities in political and health system administrative structures. The differences and similarities provided the ideal context to study common socioeconomic-cultural issues and how they impact access and use of diagnostic and treatment tools.

Sampling strategy

A purposeful sampling method was used to select participants based on these categories and also to ensure representation by age, socio-economic status, gender and geographical location. A combination of in-depth interviews (IDI) and focus group discussions (FGDs) were used to generate data for this study. Participants who participated in the in-depth interviews were different from those who participated in the FGDs. Participants for the FGDs were identified through the community leads who acted as important gate keepers in accessing these groups of participants, as they knew the area very well, were respected and trusted by the community, and were able to help with identifying potential participants. In each selected administrative region, five FGDs were undertaken with 12 participants per group and ten in-depth interviews were performed. FGDs were meant to capture a collective opinion over issues concerning diagnosis and treatment of TB. In depth interviews were to get a deeper opinion of individuals without interference or fear of others (Supplementary material: Interview guide for focus groups).

Ethics approval and consent to participate

The study aimed to collect views and opinions of individuals or groups of people in line with the ethical guidelines. To this end research team gained ethical approval from respective institutional and national ethics committees represented in the consortium. In the UK, approval was obtained from University Teaching and Research Ethics Committee of University of St Andrews (MD12073), Uganda: Makerere University Institutional Research Ethics Board (IRB) and National Council of Science and Technology (HS 2129), Kenya: KEMRI Scientific and Ethics Review Unit (KEMRI/RES/7/3/1) and Tanzania: KCRI and Mbeya and southern highlands zonal IRBs and National Health Research Ethics Committee (NatHREC) at NIMR headquarters (NIMR/HQ/R.8a/vol.IX/1317). In addition to ethics approvals, access permissions were sought and obtained from local government authorities and participants involved in the study gave a written consent to participate.

Data management and analysis

All the in-depth and key informant interviews and focus group discussions (FGDs) were conducted by a trained Qualitative Data Investigators who underwent a five-day training held in Kampala, Uganda, May 2016. All the data collection tools including questionnaires and interview guides were pretested and revised for clarity and accuracy prior to being used in the field. The interviews were conducted in Swahili in Kenya and Tanzania and/or English in Uganda, in the offices or health facilities depending on the group of respondents. Audio recorders were used to record all interviews with permission from the respondents. Collected data were transcribed verbatim from Swahili and translated to English before being analysed by thematic-content approach[25]. Data was coded and synthesized using both inductive and deductive techniques by the first author and reviewed by two other Qualitative Data Investigators for consistency to produce list of themes, theme models, and classification related to the

For peer review only

For peer review only - http://bmjopen.bmj.com/site/about/guidelines.xhtml

themes[26]. In keeping with the measures advised by Barbour 2001 independently, two researchers read and re-read all focus group and interview data, recording the emergent themes. The process involved careful reading of the interview transcripts for pattern recognition which were later translated to categories, subcategories in order to draw the conclusions from the data meanwhile developing themes. Themes further analysed to identify subthemes. However, in the process where there was disagreement in the themes and subthemes developed, discussion took place until agreement to ensure trustworthy of the data was achieved[27][28].

Public involvement statement

This was a people centred study involving people at grassroots up to policymakers. The views presented in the paper are views of the community groups and individuals in the study countries. This qualitative paper is the voice of the people: patients, survivors, carers, opinion leaders and policy makers. Regional and national public and policymakers' gatherings were held to share the findings of the study and policy briefs submitted to respective ministries of health.

Results

A total of 712 participants above 18 years of age participated in this study of whom 70 % were females. 65 % were from rural areas. Table 1 highlights the characteristics of the study participants. Eight categories of individuals participated including 38 Laboratory technicians, 37 medical administrators, 29 Political leaders, 42 TB patients and survivors, 36 key informants, 42 health care providers, 27 caregivers and 19 private practitioners in the private and public sectors. 108 focus group discussions (FGDs) were held among community members, TB patients and caregivers. 270 in-depth interview (IDI) including key informant interviews (KIs) were conducted [Table 1].

Respondents	Total number
Laboratory technicians	38
TB patients	32
TB survivors	10
TB patient care givers	27
Local government authorities	29
Medical administrators and DTLCs	37
Medical practitioners/clinic staff/DOT Nurses	42
Private practitioners	19
Key Informants	21
Community members	187

Table 1: Categories of respondents that participated in the study. Community members participating in FGDs and interviews constituted the largest proportion of participants.

Participants came from thirteen regions and fourteen counties with high prevalence of TB and HIV. In Tanzania the study was conducted in the following thirteen regions: Kilimanjaro, Manyara, Tanga, Mwanza, Kagera, Kigoma, Singida, Mbeya, Dodoma, Dar-es-salaam, Mtwara, Rukwa and Katavi. In

For peer review only - http://bmjopen.bmj.com/site/about/guidelines.xhtml

Kenya the study was conducted in the following fourteen counties: Taita, Taveta, Mombasa, Garrisa, Wajir, Meru, Marsabit, Makueni, Muranga, Nairobi, West Pokot, Bomet, Bongoma and Nyamira. In Uganda the study was conducted in the following regions and Sub regions including Northern region in Karamoja, Lango, Acholi, and West Nile; Eastern region in Teso, Sebei, Bugisu, Bugwere, Samia, Bunyole, Jopathola and Busoga Sub-region of Uganda; Southern region in Buganda, Buuruuli and Kooki Sub-region of Uganda; Western region in Bunyoro, Rwenzori, Ankole and Kigezi sub-region of Uganda (Fig.1).

Interview and FGD outcomes

A total of 712 interviews and FGD transcripts were obtained. Following analysis, the responses were grouped into two thematic categories: socio-cultural and socioeconomic barriers, each containing sub-themes including: knowledge of TB, diagnosis and treatment, Size, taste, and number of pills and side effects of TB treatment and Work patterns and responsibilities**.**

Socio-cultural barriers

Beliefs and use of alternative medicine: Several beliefs about TB disease, its causes and treatment existed in the community that delayed community members to seek care at health facilities. Regarding the cause of TB, some participants believed that it is caused by witchcraft. For example, one TB patient said,

> *"…Before I was diagnosed to have this disease, I had consulted more than one traditional healer. My mother believed that I was bewitched" (TB patient, 3 TZ).*

In addition to cultural beliefs about the cause of TB, a variety of beliefs about alternative medicines of TB in the community were widespread as reported by the study participants. The community members did believe that the readily available local medicines in the community have the capacity of curing TB. For example, one TB patient said, "*here is a medicine called Irubukoi and most of the people use them to cure TB and pneumonia*'' (TB Patient, 4 TZ]

Apart from the local medicine and traditional healers, some community members believe TB is caused by spirits and can be treated through prayers in churches or mosques as illustrated below:

"…There is this community of Nubis, they are our relatives, and we intermarry a lot, but they love their things to do with spirits. They believe a sick person is possessed by spirit and hence they go to their spiritual leaders for prayers, yet they have TB or HIV…" [TB Care Giver, 3 KE]

The beliefs which make people think of other ailments first when they are confronted with TB symptoms and/or make them delay seeking treatment from healthcare facilities or not seek it at all are quite prevalent amongst communities in the region. One healthcare provider shared example of how such beliefs manifest: "*we meet some clients who had non-medical treatment from home, because of the culture. There are people who tried to use herbs. They tried to treat a number of conditions with herbs. And they tend to take too long to seek treatment from healthcare facilities. An attitude like: "this one is culturally known to do it, even if you go for the medical treatment, it may not help, let's use this" [Health care provider, 1 KE]*.

Another healthcare provider said *"everyone knows about this, even children. Their mothers first buy them syrups over the counter not prescribed by the doctor and they try treatment at home. When their treatment fails or seems to be failing that is when they think of going to the clinic. When we ask what treatment they took from home, people often buy first line anti-biotic treatment-someone can buy amoxicillin, if there is no improvement, they change to another antibiotic of different name thinking that there will be improvement thereafter." [Health care provider, 2 TZ]*

For peer review only - http://bmjopen.bmj.com/site/about/guidelines.xhtml

Knowledge of TB, diagnosis and treatment: Knowledge about TB disease, causes, recognition and interpretation of signs and symptoms, and treatment was found to be low in the community members with about 50 % of respondents having some basic knowledge of TB. The low knowledge of TB was more pronounced rural communities compared to their counterparts living in the urban areas. The statements below illustrate the low knowledge on TB disease.

> *"…TB is transmitted by doing heavy jobs like farming and engaging in sexual intercourse with girls and smoking…" [TB Patient, 1 TZ].*

In contrast to the general community members, people who have suffered from TB and care givers of TB patients had high awareness of TB and basic knowledge of TB disease. The majority of TB patients and their caregivers knew basic facts about TB transmission, common signs and symptoms, and that TB is curable. In addition, some even mentioned about the association between TB and HIV. For example, one interviewee said:

> *"…TB is a chronic illness. TB symptoms are severe cough of more than two weeks, coughing the sputum which are blood mixed, fever during the night and overnight sweating.''* [TB Survivor, 2 TZ].

Despite the high awareness of TB among TB patients and their caregivers, it was noted that most TB patients and caregivers were not aware of TB symptoms before they had suffered from the disease. It was only after diagnosis and getting education from the health care providers that they got to know some basics of TB as one TB survivor commented,

> *"…I really never thought it could be TB because I was coughing, and I thought it was a normal cough or something like pneumonia. TB is not a small disease"* [TB Survivor, 3 TZ].

As a result of low awareness of TB disease, recognition and interpretation of TB symptoms and signs among community members, perceived symptom severity was what pushed them as last resort to take their patients to the health facility for diagnosis and treatment, as stated below:

> *"…First the (Tb patient) started coughing and to lost weight, we saw his body changing daily, he lost appetite, he was tired all the time, very much sweating especially at night he was complaining about chest pain. From there his condition continued to be worse, we decided to take him to hospital..." [Male Care Giver, 1 KE].*

Another caregiver alluded to this notion by stating

> *"…my son experienced regular fever and coughing for almost four weeks then we brought him to the hospital. But we did not notice its TB". We Tanzanians have a bad habit of taking things easy. We thought it was a normal cough but after four weeks…he was getting very serious, that is when we brought him to the hospital where we found it is TB"* [Female Care Giver, 2 TZ].

Size, taste, and number of pills and side effects of TB treatment: Apart from alternative medicine, beliefs and traditions, the number and size of pills was among the challenges mentioned to interfere with treatment adherence as reported by a male care giver *"…the huge size of these pills sometimes discourages patients from swallowing them…when I used to give the pills to my father he used to complain that they were too big to swallow and thus he wanted the small ones."*[Male care giver, 4 TZ] Another interviewee mentioned *"…the moment you are discovered remember you go, you undergo these 4 tablets plus the other ones 5, plus if you are co-infected more, so its pill burden…"* [TB patient,5

For peer review only - http://bmjopen.bmj.com/site/about/guidelines.xhtml

UG]. In a focus group discussion, a female survivor of TB [4 TZ] pointed out bitterness of the pills as another hindrance to taking the medicines and suggested sugar-coating the pills to make them more palatable.

The side effects associated with anti-TB drugs were pointed out as one of the major reasons as to why TB patients apparently fail to complete treatment. Drawing on their lived experience, respondents shared descriptions of the side effects of the drugs *"…All of them (patients on treatment) will complain of dizziness. Others have vomiting if they are not getting used to the medicine. Others reacted to the medicine and lost hearing. What we call neuropathy, the nerves, they will get a lot of pain-you get up in the morning and you are unable to walk. When you step on the ground you feel like you are stepping on thorns and sharp objects."* [Health care provider, 3 TZ].

Another health care provider added by saying *"…Somebody treated, declared cured, comes back with a cough. About three of them, all of them I managed to send the samples to Mulago for what we call mycology culture. All of them have shown to be having fungal diseases, which is making them weak and when they are coughing this time they don't cough like TB. The cough is severe. They get wasted in a shorter time. That fungus it is not TB. Well, the drug you use for treating TB are very difficult drug, in terms of their compromising the immunity. Now, once immunity drop to a certain point and remember the lung is damaged. And still this fungus also takes advantage of the weak immunity and the damaged lung to develop in your body."* [Healthcare provider, 4 UG]

Stigma associated with TB disease: The study found that stigma was rife among the patients, family, relatives, friends and neighbours, including perceived and enacted stigma as well as discrimination. All TB patients interviewed reported experiencing stigma and discrimination from the immediate family members, the community and even at health facilities. The following statements illustrate how TB patients were stigmatized and discriminated

"…I used to be fearful; ashamed I could not move a lot because I knew people were talking about me. I stayed in the house. I would just get outside next to my house and warm myself in the sun because I did not have visitors, they did not want to come…" [TB patient, 7 KE].

In addition, TB patients are also stigmatized to having HIV too. There was a sense in the community that if one has TB, s/he is most likely to have HIV too. Talking about this misconception, one participant said "*…most of the people know that when a person is having TB then this person is likely to have HIV/AIDS as well…[Community member, 1 TZ]*''. Another one said *"…because they appear emaciated, people might think they have HIV but some may not be having HIV they are just TB without other comorbidity…So I think people should not be afraid the tests… "* [Private practitioner, 1 TZ]

Fear of HIV testing: The policy recommending HIV test for every TB patient together with the perception that TB patient must have HIV, has created fear among people fear to go for TB testing. The community members fear that they will be asked to check for HIV status as well which eventually delays access and use of the TB diagnostic services. The following statements illustrate the fear of HIV testing in the community.

"…People fear to go to the hospital for TB diagnosis because they will be diagnosed and if they have TB, HIV will also be tested …" [TB Survivor, 6 TZ]

Socio-economic factors

For peer review only - http://bmjopen.bmj.com/site/about/guidelines.xhtml

Costs related to health facilities: A common view amongst most of respondents especially in the rural settings was that costs incurred in using health services including transportation was a significant socio-economic barrier to accessing TB diagnosis and treatment services. For example, one TB patient said "*… when you plan to come to the hospital you need to arrange for bus fair, accommodation cost. You cannot come to town and go back home the same day… It is very far*" [Female TB Patient, 13 TZ]. This view was echoed by another informant who said "*…I use Tz.shs. 2,000/= to pay for the bus transport coming to hospital and returning home…*" [Female Care Giver, 5 TZ].

"*…Sputum analysis you pay Ug.shs. 20,000/= for the gram stain that is when you are expecting a bacterial infection: and the ZN (Ziehl-Neelsen) that is now testing for the AFBs (Acid-Fast Bacilli), which is for TB, which causes TB. So, the whole test cost Ug.shs.20,000/= Ug.shs. 10,000/= for the gram stain and Ug.shs.10,000/= for the ZN.*" [TB patient, 9 UG]

Despite the costs, geographical accessibility also played a significant role in hindering community members to access to health facilities for TB diagnosis and treatment adherence. One caregiver commented "*…so from here to our hospital is a bit far since we are in the interior and especially when we have rains like this time, to travel from here to the hospital is very hard because accessibility of roads is very hard…*" [Care giver, 6 TZ].

It is the general view of respondents that their respective health care facilities are ill-equipped for TB care, as indicated by shortage of drugs to treat TB, especially Multi-Drug Resistant TB (MDR-TB) and they have to refer patients. A respondent, for example, testified:

"*… In case we don't have medicine at that time for MDR-TB you would have to transport the patient to Mulago. The problem is appropriate vehicle to transport them as couldn't travel in the public bus. We must get a pickup (truck) for them to sit and refer them. We usually refer to the national TB centre. But that is not encouraged, because you are now moving around a dangerous disease. It is easier to bring the medicine-source for the medicine and bring it here than sending the person there.*" [Healthcare provider, 5 UG].

Lack of food during treatment: Shortage of food was one the socio-economic concerns highlighted for non-adherence to TB medication. This theme recurred throughout the data set as a significant socio-economic barrier to keeping up with medication adherence. Due to lack of food, some patients skipped medication or even stopped medication. For example, one TB patient said "*...there are few times when I skipped my dose if I did not have enough food to eat, because if you take those drugs on an empty stomach, they make you feel very bad for like two three hours*". [TB Patient, 10 KE] Another TB patient also echoed this view, "*…TB medicine is a dose of six months, and you should take it every day. Others do stop using the medicines because of lack of food*'' [TB patient, 11 TZ].

Work patterns and responsibilities: Working patterns and conditions such as mining not only creates a high-risk environment for TB transmission but also prevented miners from accessing TB diagnosis as well as treatment adherence. For example, one miner commented *"…The young men working in mining areas because we are at a very high risk of contracting TB but when we fall sick, our bosses do not give us anything…" [Community member, 2 TZ].* Apart from working patterns and conditions, family responsibilities among caregivers somehow do play role in hindering caregivers' accessing TB diagnosis services and even keeping up with TB treatment adherence. This notion was shared by one village leader who stated **"***…most of the TB patients are poor, so when they feel relief even in the middle of the dose, they tend to stop and get back to work in order to earn a living…"* [Village leader, 3 TZ**].**

For peer review only - http://bmjopen.bmj.com/site/about/guidelines.xhtml

Lifestyle: The nomadic lifestyle among the Maasai tribe also poses as a socioeconomic barrier to seeking TB diagnosis and treatments services at health facilities. Moving from one place to another greatly interferes with medication adherence. *"…Maasai are living very far from the health centres because they tend to move from place to place far from town looking for the pasture for their animals…"* [TB Care Giver,8 TZ]. Alcohol consumption among the young men in the societies have also shown to be a contributing factor to non-adherence with TB medications. For example, one miner commented "…*most of the young men in this street are alcohol drinkers and this tendency disturbs the adherence to TB medicines…*'' [Community member, Miner, 3 TZ].

Discussion

The study aimed to assess and elucidate how the socioeconomic and cultural factors affect the uptake and utilisation of diagnostic and treatment tools for tuberculosis in East Africa. The study has brought to the fore the views of a diverse of group of stakeholders and unravels the similarities in socioeconomic-cultural perspectives across national borders. Most importantly, the study findings reveal the criticality of understanding the sociocultural context of service users in order for health systems to offer effective healthcare. including patients, survivors, caregivers, healthcare workers and administrators, and policymakers. Socioeconomic and cultural issues highlighted by the study: knowledge of disease signs and causes, perverted beliefs and use of alternative medicine, stigma and deterrent costs of care are fundamental and can be generalized to other disease conditions. For instance, anecdotal information shows COVID-19 has caused rise in stigmatization of cough and use of alternative medicines. The recently reported decline in TB notification could be reflecting people fearing to seek formal treatment for cough and resorting to purchasing medicines over-the-counter or using traditional herbs[29]

Lack of knowledge or correct information about TB transmission and treatment was among the prominent factors hindering timely and successful diagnosis and treatment of tuberculosis. These findings concur with what other studies have found on this topic[14,19–22,30,31]. Shortage of communication channels like radios, TVs, and internet exacerbate lack of awareness of TB and existing medical remedies among people mostly in rural areas and mining industry. Poor or no electricity supply complicate the use of TVs and computers. Misinformation which is currently rampant in the COVID-19 era has always been a hinderance to uptake medical interventions and can only be counteracted by proactive sharing of facts.

Access and use of health technologies is further compromised by poor transport infrastructure, high illiteracy rate, and lack of well-equipped health care facilities and specialized health care providers[32,33]. Analysis of the health system barriers (first part of this study) points to the centralized implementation model as the most cost-effective of ensuring maximal uptake and utilisation of high-tech diagnostic technologies. However, this can only be effective if the governments invested in good transport infrastructure connecting rural and urban areas to ensure timely sample referral and delivery of diagnostic samples. Investing in information communication technologies would not only ensure higher awareness of TB and its care services but also communicating laboratory results to patients once tests are completed. Hiring and retaining well trained healthcare practitioners and giving them refresher training on novel health technologies cannot be more emphasized.

The current tuberculosis treatment regimen is a combination of four antibiotics for six months in drug sensitive TB and more months for drug resistant TB[34]. Presence of comorbidity like HIV worsens the pill burden. The pill burden (size and number of pills) was highlighted by participants as major hinderance to adherence to treatment. Side effects such as vomiting, and loss of hearing aggravated the problem. This calls for medicines developers to find innovative ways of combining drugs into single

For peer review only - http://bmjopen.bmj.com/site/about/guidelines.xhtml

pills and less bitter to increase palatability. Finding fewer toxic drugs or combinations of drugs should be the main agenda of drug manufacturers and clinical trials. Increasing patient – practitioner communication and interaction has been suggested as one of the ways to manage and mitigate side effects caused by medicines and ensuring treatment adherences[35]. Patients should be well informed about the side effects, pill burden as well as supportive nutritional foods they need while on treatment.

Our study found stigma to TB infection was mostly associated with HIV infection, since the general community perception is that anyone testing positive for TB is also HIV infected. As much as the perception is incorrect because HIV-TB co-infection is estimated at 40% in East Africa, it shapes the attitudes in the negative direction[36]. This contrasts the TB stigma in West Africa where TB is associated with ethnic or family curse, being grossly unhygienic and very contagious so much that even healthcare practitioners shun working with TB patients[37]. Consequently, stigma make people more inclined to keeping their ailment a secret and reduces willingness to seek medical care. This in turn stops or delays access to appropriate diagnostic and treatment services[17,18,38,39]. We believe poor understanding of the causes and signs of disease like TB creates a knowledge gap that people tend to fill with superstition and stigma. Those purposed to have knowledge such as healthcare practitioners, their stigma may be driven by lack of personal protective equipment (PPE) for which they ensure safety by avoiding patients with contagious diseases like TB. Sensitisation of both community and healthcare practitioners coupled with provision of appropriate PPE would go a long way in resolving the stigma issue.

Beliefs and myths about the causes and transmission for TB including being related to witchcraft, family curse and having a long Uvula ***(known as Kilimi in Swahili both in TZ and KE)*** as cause of recurrent coughing raised stigma and discrimination of TB patients. As a result, the patient pathway to care is complicated as most patients resort to seeking remedy from traditional healers, palatine uvula cutters before going to the formal healthcare centres. These kinds of behaviours and beliefs have been reported in other studies in done in Tanzania[38] and in other African countries[40]. Action follows belief and so someone who believes witchcraft is the cause of TB will by default consult a traditional healer or someone with spiritual powers first, which prolongs the pathway to diagnosis and treatment at formal healthcare facilities[35,38].

Our study is the first large qualitative study combining information from three Eastern Africa countries. It has highlighted the critical impact socioeconomic and cultural barriers have on uptake and utilisation of diagnostic and treatment technologies. Drawing from the findings it appears the largest "roadblock" to uptake and utilisation of diagnostic and treatment tools occurs at the primary point of access in the patient pathway. This is the point between feeling poorly and getting diagnosis. It therefore makes more sense for most investment to be made at this primary point of access in order to shorten the path to access of healthcare services, which in turn would increase uptake, and utilisation of health technologies (Fig. 2)

Strengths and Limitations

This study brought together diverse group of stakeholders ranging from community members at village level to practitioners and policy makers at regional and national level. Their views give an enriched perspective of how socioeconomic and cultural barriers affect uptake and utilisation of health technologies within the health system. Triangulation of data sources strengthens the validity of our results. The findings represent voices of real people and can be generalised to other diseases in as far as healthcare access is concerned. We note that views of 712 participants may not be representative of everyone in the study countries. Reliance of participant's ability to recall events of their previous interactions with the health care system raises risk of inaccuracy in the data collected. Quite importantly, however, is that our interviewers were well-trained with experience in conducting social science studies.

For peer review only - http://bmjopen.bmj.com/site/about/guidelines.xhtml

Conclusion and Recommendations

Our study has shown that socioeconomic and cultural factors are important barriers to uptake and utilisation of diagnostic and treatment tools. While our study was done in the context of tuberculosis, we believe these challenges are fundamental and can apply to any other disease. Resolving awareness/knowledge gaps, beliefs, stigma and improving socioeconomic welfare of affected communities is an antidote that cuts across many disease conditions. We recommend future socioeconomic – cultural interventional studies with monitoring and evaluation component to assess and quantify impact of the interventions. Learning from end users by diagnostics and medicines developers will enable them to develop effective diagnostic and treatment tools. Social protection programmes to people working in high-risk industries like mining and interventions to reduce use of dirty sources of energy like firewood, charcoal, coal etc are critical for reducing the burden of respiratory diseases like TB. Focusing investment at the primary point of healthcare access is critical to maximising uptake and utilisation of diagnostic and treatment tools.

Conflicts of Interest

Authors declare no conflict of interest.

Data sharing statement

Extra is available upon request and meeting the confidentiality and use of data requirements signed between authors and participants. Email ws31@st-andrews.ac.uk to register your request.

Acknowledgements

The authors acknowledge the contributions of Dr Ewan Chirnside and Prof Gibson Kibiki in the initial study conception, design and implementation of the study. Prof Kibiki also played a big role in linking the TWENDE consortium to East African Health Research Commission (EAHRC). As a result of his effort, EAHRC officially adopted TWENDE as a research consortium for generating evidence needed for translation of health technologies to policy and practice in the East African Community. Special memories of Dr Alphonse Okwera who was the Uganda country PI for the TWENDE study and sadly passed away before this manuscript was written. Thanks to Ms Norah Owaraga who coordinated the training of qualitative researchers in the TWENDE consortium. We also acknowledge Dr. Veronica O'Carroll and Dr Stella-Neema for qualitative expertise they to proofread the manuscript that improved the cogency of the manuscript.

Funding

The study was funded by the European and Developing Countries Clinical Trials Partnership (EDCTP), grant TWENDE-EDCTP-CSA-2014-283.

Author contributions

Study design: WS, SHG, NEN, EA, BTM, MJ; Data collection and collation: EFM, FO, ES, BM, SM, HM, KK, AL, IM; Drawings: AA & WS; Data analysis: EFM, FO, ES, HM; Manuscript drafting: EFM, ES, FO, WS; Manuscript review: All co-authors.

References

1 World Health Organization. Are Updated Every Year . for the Tuberculosis. 2019. https://www.who.int/tb/publications/global_report/en/

2 WHO. Global TB Report. Geneva 27, Switzerland: 2014. ISBN 978 924 1564809

For peer review only - http://bmjopen.bmj.com/site/about/guidelines.xhtml

3 Murray CJL, Ortblad KF, Guinovart C, *et al.* Global , regional , and national incidence and mortality for HIV , tuberculosis , and malaria during 1990 – 2013 : a systematic analysis for the Global Burden of Disease Study 2013. 2014;**6736**:1–66. doi:10.1016/S0140-6736(14)60844-8

4 Lönnroth K, Castro KG, Chakaya JM, *et al.* Tuberculosis 1 Tuberculosis control and elimination 2010 – 50 : cure , care , and social development. *Lancet* 2015;**375**:1814–29. doi:10.1016/S0140-6736(10)60483-7

5 Zumla A, Gant V, Bates M, *et al.* Rapid diagnostics urgently needed for killer infections. *Lancet Respir Med* 2013;**1**:284–5. doi:10.1016/S2213-2600(13)70099-7

6 Lawn SD, Mwaba P, Bates M, *et al.* Advances in tuberculosis diagnostics: The Xpert MTB/RIF assay and future prospects for a point-of-care test. *Lancet Infect Dis* 2013;**13**:349–61. doi:10.1016/S1473-3099(13)70008-2

7 Parsons LM, Somoskövi Á, Gutierrez C, *et al.* Laboratory diagnosis of tuberculosis in resource-poor Countries: Challenges and opportunities. *Clin Microbiol Rev* 2011;**24**:314–50. doi:10.1128/CMR.00059-10

8 Pandey V, Singh P, Singh S, *et al.* SeeTB : A novel alternative to sputum smear microscopy to diagnose tuberculosis in high burden countries. 2019;:1–10. doi:10.1038/s41598-019-52739-9

9 Zijenah LS, Zijenah S. The World Health Organization Recommended TB Diagnostic Tools Organization Recommended TB The World Health Diagnostic Tools. doi:10.5772/intechopen.73070

10 Boehme CC, Nicol MP, Nabeta P, *et al.* Feasibility, diagnostic accuracy, and effectiveness of decentralised use of the Xpert MTB/RIF test for diagnosis of tuberculosis and multidrug resistance: A multicentre implementation study. *Lancet* 2011;**377**:1495–505. doi:10.1016/S0140-6736(11)60438-8

11 Nahid P, Pai M, Hopewell PC. Advances in the diagnosis and treatment of tuberculosis. *Proc Am Thorac Soc* 2006;**3**:103–10. doi:10.1513/pats.200511-119JH

12 Chimnani J, Kamunyori J, Tuddenham J, *et al.* Tanzania : TB and Leprosy Logistics System Assessment. 2011.

13 Albert H, Bwanga F, Mukkada S, *et al.* Rapid screening of MDR-TB using molecular Line Probe Assay is feasible in Uganda. *BMC Infect Dis* 2010;**10**. doi:10.1186/1471-2334-10-41

14 Vries SG De, Cremers AL, Heuvelings CC, *et al.* Series Tuberculosis in hard-to-reach populations 1 Barriers and facilitators to the uptake of tuberculosis diagnostic and treatment services by hard-to-reach populations in countries of low and medium tuberculosis incidence : a systematic review of qualit. 2017;**3099**:1–16. doi:10.1016/S1473-3099(16)30531-X

15 Watermeyer J, Penn C. Community perspectives on tuberculosis care in rural South Africa. 2018;:1–9. doi:10.1111/hsc.12637

16 Ngamvithayapong J, Winkvist A, Diwan V. High AIDS awareness may cause tuberculosis patient delay : results from an HIV epidemic area , Thailand. 2000;:1413–9.

17 Courtwright A. Tuberculosis and Stigmatization : Pathways and Interventions. ;**125**:34–42.

18 Jittimanee SX, Nateniyom S, Kittikraisak W, *et al.* Social Stigma and Knowledge of Tuberculosis and HIV among Patients with Both Diseases in Thailand. 2009;**4**:6–12.

For peer review only

For peer review only - http://bmjopen.bmj.com/site/about/guidelines.xhtml

doi:10.1371/journal.pone.0006360

19 Kipp AM, Pungrassami P, Stewart PW, *et al.* Study of tuberculosis and AIDS stigma as barriers to tuberculosis treatment adherence using validated stigma scales. 2011;**15**:1540–5.

20 Sima BT, Belachew T, Abebe F. Knowledge , attitude and perceived stigma towards tuberculosis among pastoralists ; Do they differ from sedentary communities ? A comparative cross-sectional study. 2017;:1–17.

21 Royce Rachel A. Americans in Three Southeastern Counties in the United States : 2017;:1–21. doi:10.1007/s40615-015-0200-1.Tuberculosis

22 Gelaw SM. Socioeconomic Factors Associated with Knowledge on Tuberculosis among Adults in Ethiopia. 2016;**2016**.

23 Stuckler D, Basu S, Mckee M, *et al.* Mining and Risk of Tuberculosis in Sub-Saharan Africa. 2011;**101**:524–30. doi:10.2105/AJPH.2009.175646

24 Crush J, Williams B, Gouws E, *et al.* Migration and HIV / AIDS in South Africa. 2005;**22**.

25 Graneheim UH, Lundman B. Qualitative content analysis in nursing research: Concepts, procedures and measures to achieve trustworthiness. *Nurse Educ Today* 2004;**24**:105–12. doi:10.1016/j.nedt.2003.10.001

26 Piergiorgio Corbetta. *Social Research : Theory , Methods and Techniques The Use of Documents*. London: : Sage Publications 2003. doi:http://dx.doi.org/10.4135/9781849209922.n11

27 Barbour RS. Checklists for improving rigour in qualitative research: a case of the tail wagging the dog? *Bmj* 2001;**322**:1115–7. doi:10.1136/bmj.322.7294.1115

28 Creswell, Data Analysis and Representation. Sage publications Inc. 2017

29 WHO 2020. Infection Prevention and Control guidance for Long-Term Care Facilities in the context of COVID-19. *Interim Guid World Heal Organ* 2020;:1–5.

30 Kigozi NG, Heunis JC, Engelbrecht MC, *et al.* Tuberculosis knowledge , attitudes and practices of patients at primary health care facilities in a South African metropolitan : research towards improved health education. 2017;:1–8. doi:10.1186/s12889-017-4825-3

31 Asres M, Gedefaw M, Kahsay A, *et al.* Patients' delay in seeking health care for tuberculosis diagnosis in east gojjam zone, northwest Ethiopia. *Am J Trop Med Hyg* 2017;**96**:1071–5. doi:10.4269/ajtmh.16-0892

32 Ereso BM, Yimer SA, Gradmann C, *et al.* Barriers for tuberculosis case finding in Southwest Ethiopia: A qualitative study. *PLoS One* 2020;**15**:1–20. doi:10.1371/journal.pone.0226307

33 Alves JC de F. Socioeconomic-cultural barriers delaying the tuberculosis diagnosis. *J Nurs UFPE line* 2016;**10**:4021–7. doi:10.5205/1981-8963-v10i11a11485p4021-4027-2016

34 WHO 2017. *Treatment of tuberculosis.* Switzerland: 1954. ISBN 978-92-4-155000-0%0A©

35 Boru CG, Shimels T, Bilal AI. Factors contributing to non-adherence with treatment among TB patients in Sodo Woreda, Gurage Zone, Southern Ethiopia: A qualitative study. *J Infect Public Health* Published Online First: 2017. doi:10.1016/j.jiph.2016.11.018

36 Bonnington O, Wamoyi J, Ddaaki W, *et al.* Changing forms of HIV-related stigma along the HIV care and treatment continuum in sub-Saharan Africa : a temporal analysis. 2017;:1–6. doi:10.1136/sextrans-2016-052975

For peer review only - http://bmjopen.bmj.com/site/about/guidelines.xhtml

37 Dodor EA, Kelly SJ. Manifestations of tuberculosis stigma within the healthcare system: The case of Sekondi-Takoradi Metropolitan district in Ghana. *Health Policy (New York)* 2010;**98**:195–202. doi:10.1016/j.healthpol.2010.06.017

38 Said K, Hella J, Mhalu G, *et al.* Diagnostic delay and associated factors among patients with pulmonary tuberculosis in Dar es Salaam, Tanzania. *Infect Dis Poverty* 2017;**6**:1–10. doi:10.1186/s40249-017-0276-4

39 de Vries SG, Cremers AL, Heuvelings CC, *et al.* Barriers and facilitators to the uptake of tuberculosis diagnostic and treatment services by hard-to-reach populations in countries of low and medium tuberculosis incidence: a systematic review of qualitative literature. *Lancet Infect Dis* 2017;**17**:e128–43. doi:10.1016/S1473-3099(16)30531-X

40 Amo-adjei J, Kumi-kyereme A. Myths and misconceptions about tuberculosis transmission in Ghana. 2013.

Figure legends

Figure 1: The map showing the surveyed regions and counties in three East African Community Countries (EAC).

Figure 2: Diagrammatic representation of the impediments of access, uptake and utilisation of diagnostic and treatment tools in the patient pathway. Most hinderance is experienced at the primary level where the patient first seeks healthcare intervention during their ailment. The path is not straight reflecting the different barriers and routes the patient goes through to get- or adhere to- care.

For peer review only

For peer review only - http://bmjopen.bmj.com/site/about/guidelines.xhtml

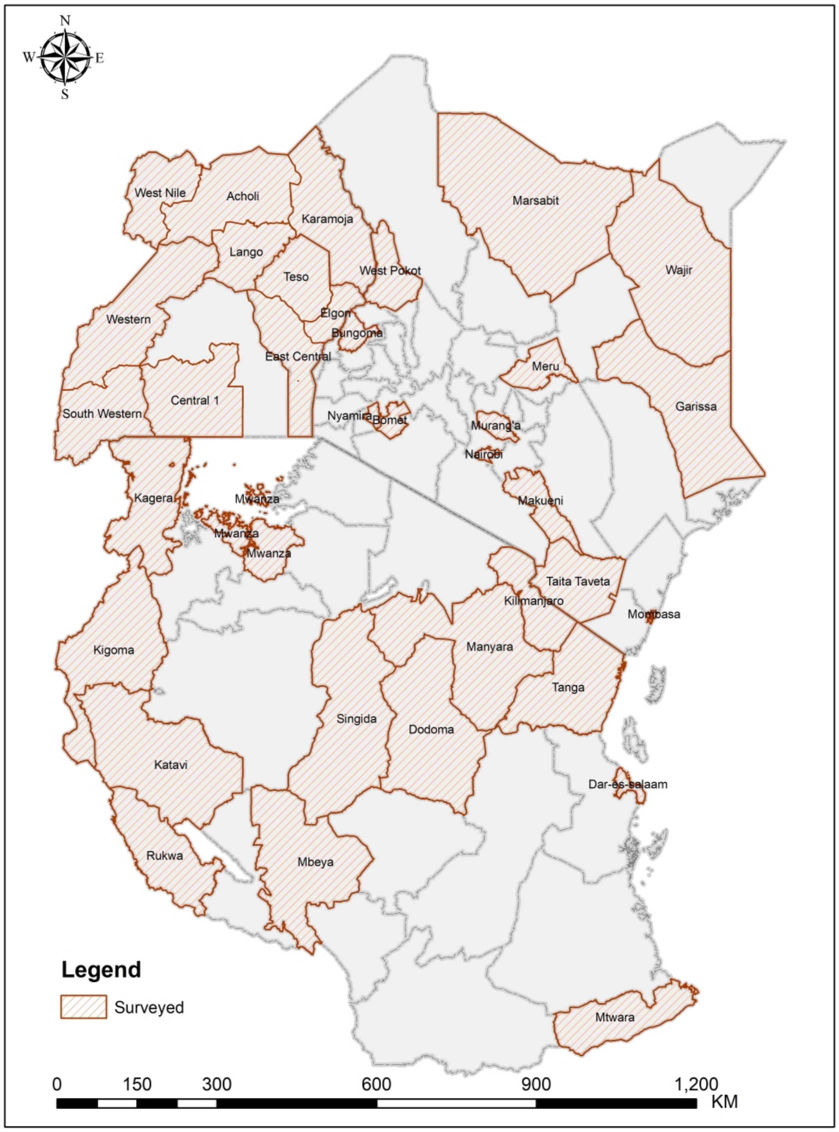

Figure 1: The map showing the surveyed regions and counties in three East African Community Countries (EAC).

215x279mm (500 x 500 DPI)

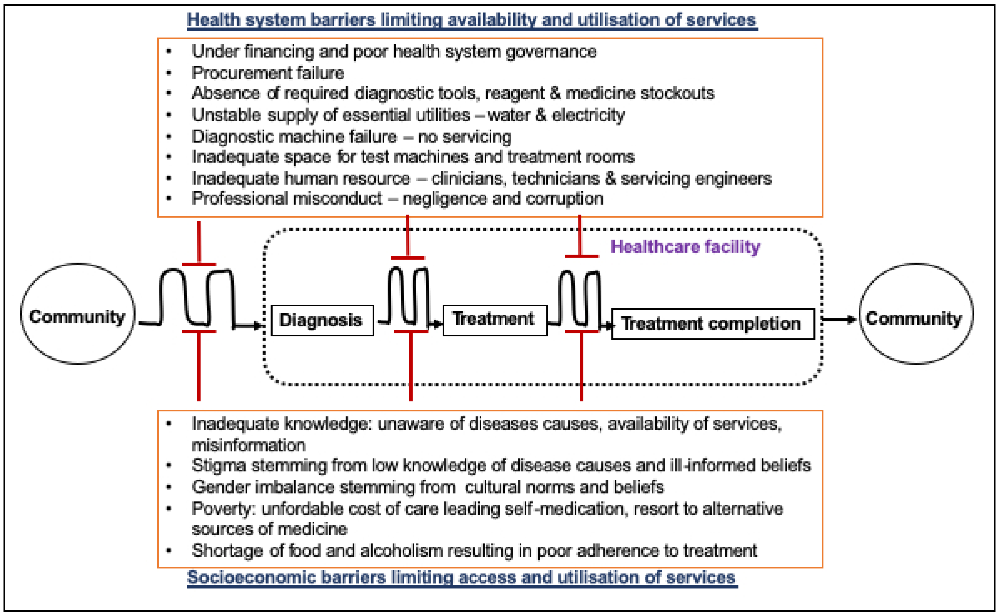

Figure 2: Diagrammatic representation of the impediments of access, uptake and utilisation of diagnostic and treatment tools in the patient pathway. Most hinderance is experienced at the primary level where the patient first seeks healthcare intervention during their ailment. The path is not straight reflecting the different barriers and routes the patient goes through to get- or adhere to- care.

281x174mm (54 x 54 DPI)

Tuberculosis: Working together to Empower Nations' Diagnostic Effort (TWENDE)

Assessing the coverage and usage of Xpert MTB/RIF & Line Probe Assay services in Tanzania, Kenya and Uganda

Interview Guide for Focus Groups & Focus Group Discussions

1.1 Opening Lines (after informed consent has been reviewed and participant has agreed)

In this Focus Group Discussion, I would like to hear your views about the '*stigma related to tuberculosis*'; '*diagnosis and treatment of tuberculosis*'. You are an expert in this subject and so feel free to share your views among each other however you wish. I may minimally interrupt your discussion and ask some questions which are coming from your comments, which are interested in order to move the discussion along, but will try and keep my interruptions very minimum. Please feel free to elaborate on anything you feel is important for me to know about. I am interested in all what you consider the negative and positives in diagnosis and treatment also what you perceive as a stigma on TB in Tanzania. Remember, there is no any right or wrong answers and your contributions are so valuable. Please feel free to discuss openly and freely about what you know on the state of stigma, diagnosis and treatment of TB among self, family, community, and national here in Tanzania.

1.2 Initial Identifying Statement of the Facilitator

So let us begin [*press play on audio recorder*]: "It is [*date*] and I am sitting in [*place*] with [*participant's group name*]. We are going to converse about their views about the topic of ''stigma *related to tuberculosis*''; ''*diagnosis and treatment of TB*" in [name district].

1.3

- In your experiences, how accessible are TB diagnostic laboratories and treatment centres in [name district]?
  - [*probe*] In your opinions, in general, what is the state of TB diagnosis and treatment services in [name district]?
  - [*Probe*]When do you go for your check-ups when you feel you have TB symptoms?
  - [*Probe*]Do you pay for TB diagnosis or treatment?
    [*Probe*]What are your recommendations, to make things better in terms of TB diagnosis and treatment services in [name district]?
- In your experiences, are TB patients in general stigmatised here in your community in [name ethnic group, village, district, region, Tanzania]?
  - If yes, how so? Why do you think so?
  - [*Probe*] What is your recommendation on how stigmatisation of TB patients may be reduced?

For peer review only - http://bmjopen.bmj.com/site/about/guidelines.xhtml

1.4 Conclusion of Interview

Thank you so much group members for taking the time to discuss with me today. I learned a great deal from this discussion, and I really appreciate your willingness to share your views, insights and opinions with me and by extension the TWENDE project.

[*Allow respondents to comment if he wishes.*]

For peer review only

For peer review only - http://bmjopen.bmj.com/site/about/guidelines.xhtml

SRQR Reporting checklist

No.	Topic	Item
	**Title and Abstract**	
S1	Tittle	Found on page 1 Line 1-2 reflecting the methodology and rationale of the study
S2	Abstract	Found on page 2 Lines 47-72 stating the problem, the methodology, findings and conclusion.
	**Introduction**	
S3	Problem formulation	Found on page 3 Lines 93-120 giving background to the problem and what is known about it.
S4	Purpose or research question	Found on page 3 Lines 122-128 stating the rationale and the question the researchers set out to answer.
	**Methods**	
S5	Qualitative approach and research paradigm	Page 3 Lines 131-137 stating the qualitative approach and the temporal context of when the research was conducted.
S6	Researcher characteristics and reflexibility	Page 4 Lines 138-143 describing the study design, participants and the research team.
S7	Context	Page 4 Lines 144-150 highlighting the important context within which the study was conducted. Further context is given on page 7 Lines 215-224.
S8	Sampling strategy	Page 4 Lines 152-163 describing the strategy used in sampling.
S9	Ethical issues pertaining to human subjects	Page 4 Lines 164-173 stating rationale for ethics and the ethical permissions obtained to conduct the study.
S10	Data collection methods	Page 4 Lines 177-196 outlining how data was collected.
S11	Data collection instruments and technologies	Page 4 Line 182 stating audio recording of interviews using audio recorders.
S12	Units of study	Page 4 Lines 160-163 states the number of people who participated in focus groups and how they were selected.
S13	Data processing	Page 4-5 Lines 182-189 data transcribing, coding and theme drawing
S14	Data analysis	Page 5 Lines 190-194 – further analysis of the themes to generate sub-themes.
S15	Techniques to enhance trustworthiness	Page 5 Lines 196-200 highlighting public involvement in the study.
	**Results/findings**	
S16	Synthesis and integration	Page 5-10 Lines 204-408 stating finding of the study
S17	Links to empirical data	Not applicable as all finding are presented in the results.
	**Discussion**	
S18	Integration with prior work, implications, transferability & contributions to the field	Found on Page 10-11 Lines 412-484, Page 12 Lines 496-508
S19	Limitations	Found on Page 11 Lines 486-495
	**Other**	
S21	Conflicts of interest	Page 12 Line 510
S21	Funding	Page 12 Line 529-520

For peer review only
